# Feasibility and acceptability to use a smartphone-based manikin for daily longitudinal self-reporting of chronic pain

**DOI:** 10.1177/20552076231194544

**Published:** 2023-08-16

**Authors:** Syed Mustafa Ali, David A Selby, Darryl Bourke, Ramiro D Bravo Santisteban, Alessandro Chiarotto, Jill Firth, Ben James, Ben Parker, William G Dixon, Sabine N van der Veer

**Affiliations:** 1 Centre for Epidemiology Versus Arthritis, Division of Musculoskeletal and Dermatological Sciences, Manchester Academic Health Science Centre, 5292 The University of Manchester, Manchester, UK; 2 Centre for Health Informatics, Division of Informatics, Imaging and Data Science, Manchester Academic Health Science Centre, 5292 The University of Manchester, Manchester, UK; 3 German Research Center for Artificial Intelligence (DFKI), Kaiserslautern, Germany; 4 Department of General Practice, Erasmus MC, University Medical Center, Rotterdam, the Netherlands; 5 Department of Health Sciences, Faculty of Science, VU University, Amsterdam Movement Sciences, Amsterdam, the Netherlands; 6 Integrated Care Centre, Pennine MSK Partnership, Oldham, UK; 7 579576 uMotif, London, UK; 8 Kellgren Centre for Rheumatology, NIHR Manchester Biomedical Research Centre, 5293 Manchester University NHS Foundation Trust, Manchester, UK; 9 NIHR Manchester Biomedical Research Centre, 5293 Manchester NHS Foundation Trust, Manchester, UK; 10 5293 Northern Care Alliance NHS Foundation Trust, Salford, UK

**Keywords:** Chronic pain, pain measurement, mobile applications, engagement, feasibility and acceptability

## Abstract

**Background:**

As management of chronic pain continues to be suboptimal, there is a need for tools that support frequent, longitudinal pain self-reporting to improve our understanding of pain. This study aimed to assess the feasibility and acceptability of daily pain self-reporting using a smartphone-based pain manikin.

**Methods:**

For this prospective feasibility study, we recruited adults with lived experience of painful musculoskeletal condition. They were asked to complete daily pain self-reports via an app for 30 days. We assessed feasibility by calculating pain report completion levels, and investigated differences in completion levels between subgroups. We assessed acceptability via an end-of-study questionnaire, which we analysed descriptively.

**Results:**

Of the 104 participants, the majority were female (*n* = 87; 84%), aged 45-64 (*n* = 59; 57%), and of white ethnic background (*n* = 89; 86%). The mean completion levels was 21 (± 7.7) pain self-reports. People who were not working (odds ratio (OR) = 1.84; 95% confidence interval (CI), 1.52-2.23) were more likely, and people living in less deprived areas (OR = 0.77; 95% CI, 0.62-0.97) and of non-white ethnicity (OR = 0.45; 95% CI, 0.36-0.57) were less likely to complete pain self-reports than their employed, more deprived and white counterparts, respectively. Of the 96 participants completing the end-of-study questionnaire, almost all participants agreed that it was easy to complete a pain drawing (*n* = 89; 93%).

**Conclusion:**

It is feasible and acceptable to self–report pain using a smartphone–based manikin over a month. For its wider adoption for pain self–reporting, the feasibility and acceptability should be further explored among people with diverse socio–economic and ethnic backgrounds.

## Introduction

Chronic pain is prevalent and has an impact on individuals and society.^1–3^ Chronic pain, previously considered solely a symptom of other long-term conditions, has recently been acknowledged as a long-term condition in its own right.^4–6^ Chronic pain deteriorates people's physical and mental health,^7^ which in turn causes disability that results in lower productivity, increased work absenteeism,^8^ impaired social functioning,^9^ and poor quality of life.^10^ Chronic pain also has substantial economic costs for society in the form of healthcare utilisation and reduced work productivity.^11–14^

Despite the importance and impact of chronic pain, detailed insights into its prevalence,^1^ aetiology,^15^ and treatment remain largely unknown.^16,17^ Also, various organisations, including the Agency for Health Care Policy and Research and the American Pain Society, indicated that management of pain continues to be suboptimal.^18–20^ Measuring pain more frequently and longitudinally in representative populations would enable clinicians, patients and researchers to track the course of pain and quantify treatment effects,^21^ thereby enhancing our knowledge and improving the management of pain.^22^

Currently available pain self-reporting tools, however, may not be suitable for frequent, longitudinal pain measurement, especially when wanting to capture multiple or more complex aspects of pain, e.g. location-specific pain intensity or quality, or spatial or temporal patterns of pain. For example, the McGill Pain Questionnaire is one of the most widely used tools but takes around 10 min to complete,^23,24^ implying a substantial data collection burden for people if asked to complete it often and an additional challenge of remembering to fill in if using a pen-and-paper version. Furthermore, completing self-report questionnaires may be more challenging for specific groups because of barriers related to age, language or cognitive abilities.^25^ One study, for example, found that pain was underreported by older adults compared to their younger counterparts.^26^ Similarly, current pain self-report tools may not adequately account for the influence of cultural background on people's pain reporting needs and behaviour.^27,28^ Lastly, an expert working group of the European Association for Palliative Care ranked location, intensity and temporal pattern as the most important dimensions for measuring pain.^29^ However, a study in palliative care settings concluded that pain assessment tools seldom covered these dimensions adequately.^29^

Digital pain manikins, also called pain drawings or pain body maps, may address some of the shortcomings of current pain self-reporting tools to measure bodily extent of pain.^30–32^ Pain manikins are human body-shaped figures on which people shade or select painful areas.^33^ Many have recognised the potential of pain manikins in general and of their digital counterparts in particular,^32,34–37^ citing potential benefits such as improved participation rates in population- level surveys, improved engagement of people with lower literacy levels, reduced survey administration cost and enabling automated calculation of pain summary scores. In addition, digital manikins are increasingly embedded in smartphone apps.^38^ And because smartphones are integrated into people's daily lives,^39^ this opens up possibilities of more frequent and longitudinal pain self-reporting.

A 2022 systematic literature review on the use of digital pain manikins for research data collection, however, found that only five of 17 included studies collected pain manikin data longitudinally, with a maximum duration of no more than 5 days.^40^ It, therefore, remains largely unknown if digital manikins could address the need for frequent, longitudinal and representative pain self-assessment. Therefore, we aimed to assess the feasibility and acceptability of a smartphone-based pain manikin for frequent and longitudinal self-reporting of pain and explore whether feasibility differed between subgroups.

## Methods

This was a prospective feasibility study where people suffering from one or more painful musculoskeletal painful condition self-reported their pain for 30 days using a smartphone-based pain manikin.

### Participant eligibility and recruitment

We invited potential participants through four rheumatology departments at the National Health Service (NHS) sites in Greater Manchester (United Kingdom) and via national and regional patient social media groups. People could express their interest after reading the participant information sheet and study flyer and watching the study orientation video. The participant information sheet explained the purpose of the feasibility study, clarified the study would not directly impact or benefit their pain treatment and suggested possible future developments the research might inform (e.g. embedding the manikin in a pain management intervention for improving self-management or clinical decisions). Adults (aged 18 and above) with a clinical diagnosis of at least one of three musculoskeletal painful conditions (i.e. fibromyalgia, rheumatoid arthritis or osteoarthritis) and with daily access to the internet and an Android smartphone or tablet (version Oreo 8.0 and above) were eligible to take part. One researcher (SMA) recruited all participants from June to October 2021 and screened people by telephone for eligibility and obtained written informed consent.

### Data collection procedure

**Figure 1.**
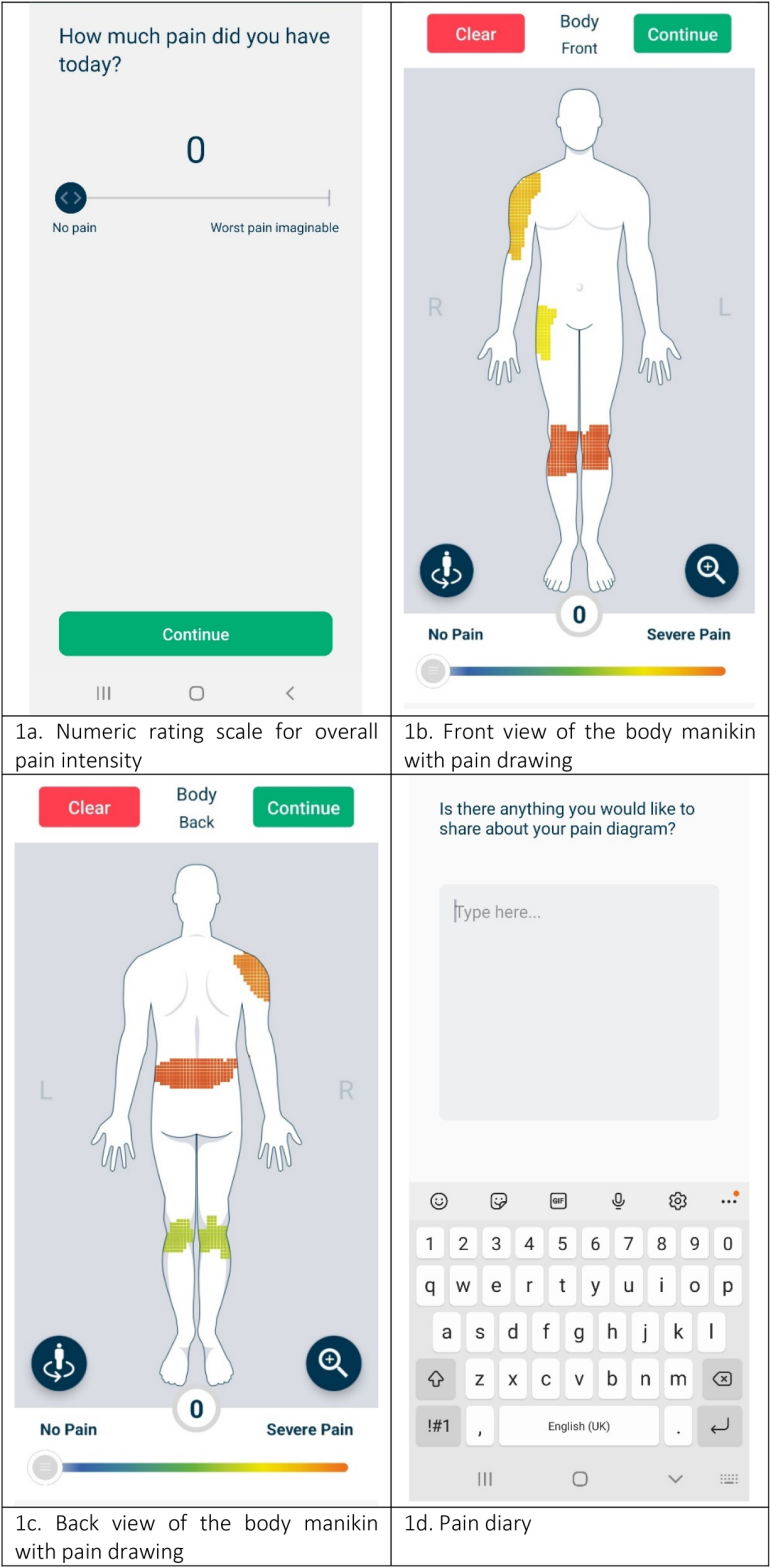
Screenshots of the Manchester Digital Pain Manikin app (copyright University of Manchester).

Prior to the start of the pain self-reporting period, participants were invited to self-complete an online questionnaire to capture their baseline characteristics (Supplementary Material 1). Upon completing the baseline questionnaire, we emailed participants a user guide (Supplementary Material 2) and offered them an optional session via Zoom to help them with downloading, installing and using the Manchester Digital Pain Manikin app. This app, developed by a technology partner, was an improved version of a prototype that we had developed with input from patient representatives; previous evaluations had focused on general usability and selecting the granularity of the underlying grid.^41^ We asked participants to complete a daily pain manikin report on the app for a pain self-reporting period of 30 days using their own Android-compatible smartphones or tablets, guided by in-app instructions and reminded by an automated notification every night at 8 PM. A daily manikin report included a single overall pain question, a two-sided two-dimensional pain drawing and a free text pain diary (Figure 1(a) to (d)). Participants could draw their pain on the front and back view of the two-dimensional, gender-neutral manikin. They could zoom in by selecting pre-specified body areas from a list. Drawing involved selecting a pain intensity using the sliding scale at the bottom of the screen and shading painful areas directly on the manikin. In case of a pain-free day, we instructed participants to submit an empty manikin report. There was no in-app archive available for participants to view previously submitted pain drawings, but we emailed participants a summary of their pain reports at the end of their study period.

At the end of the pain reporting period, we invited participants to self-complete an online acceptability questionnaire to capture their views on the app (Supplementary Material 3), including its usability, usefulness and their willingness to use it for pain self-reporting in future studies. We developed this questionnaire based on the Technology Acceptance Model,^42^ the QQ-10^43^ and a systematic review on digital pain manikins.^38^ Upon completing the questionnaire, participants received a £20 gift voucher to thank them for taking part.

### Data analysis

We used the mean (± standard deviation (SD)) pain report completion level as the primary outcome for assessing the feasibility of frequent, longitudinal pain self-reporting, which is consistent with one of the four engagement constructs, i.e. objective engagement – higher completion level indicates greater feasibility.^44^ We defined each participant's completion level as the number of days where they submitted at least one manikin report, with the maximum completion level being 30 days. Empty manikin reports were counted towards completion levels as we instructed participants to submit these on pain-free days. We visualised the distribution of participants’ completion levels in the form of dot plots. For calculating secondary outcomes related to feasibility, we divided the pain self-reporting period into 4 weeks of 7 days of pain self-reporting, with week 1 starting on the day of the first report and week 4 ending on day 28. We then calculated the number of participants with at least (a) one complete week (i.e., seven consecutive days with a manikin report), (b) two or more than two manikin reports in each of the four weeks, and (c) five or more than five manikin reports in each of the four weeks.

We performed multivariate binomial regression analyses to investigate differences in completion levels between subgroups. Subgroups were based on musculoskeletal condition (rheumatoid arthritis, osteoarthritis, fibromyalgia or more than one of these) and on demographic characteristics that previous studies identified as factors potentially affecting engagement with pain reporting and/or with digital technology (i.e. age, gender, ethnicity, socio-economic status, employment status).^45,46^ For each subgroup, we developed a model adjusting for all other characteristics, presenting results as odds ratios (ORs) with a 95% confidence interval (CI).

Lastly, we analysed participants’ responses to the acceptability questionnaire as frequencies and percentages.

We used RStudio 2022.02.3 (Build 492) software for all data analyses.

## Results

### Participant characteristics

Of the 131 eligible people consenting to take part, 104 submitted at least one manikin report. Table 1 shows that the majority were aged 45 or older (*n* = 83; 80%), female (*n* = 87; 84%), identified as white British (*n* = 87; 84%), had only one painful musculoskeletal condition (*n* = 87; 84%) and almost half of them had lived experience of pain for over 10 years (*n* = 53; 51%). Supplementary Table 1 (Supplementary File 1) presents the information we had available on the 27 people who consented but did not submit a manikin report.

**Table 1.** Characteristics of study participants (total *n* = 104).

Characteristic	Categories	Number (percentage)
Age	44 or younger	21 (20)
	45–64	59 (57)
	65 or older	24 (23)
Gender	Male	17 (16)
	Female	87 (84)
Ethnicity^a^	White	87 (84)
	Non-white	17 (16)
Employment status^b^	Employed	45 (43)
	Not working	50 (48)
	Other	9 (9)
Index of multiple deprivation decile^c^	1 to 3 (most deprived)	53 (51)
	4 to 6	25 (24)
	7 to 10 (least deprived)	22 (21)
	Missing	4 (4)
Musculoskeletal condition	Osteoarthritis (OA)	31 (30)
	Rheumatoid arthritis (RA)	31 (30)
	Fibromyalgia	25 (24)
	More than one condition	17 (16)
Pain experience (in years)	Less than a year	3 (3)
	1–3 years	16 (15)
	4–10 years	32 (31)
	More than 10 years	53 (51)

^a^ ‘White’ included all people identified as being from white ethnic backgrounds, which included mixed/multiple ethnic groups; the remaining ethnicities were recoded as ‘Non-white’.

^b^ ‘Employed’ included employed and self-employed; ‘Not working’ included unemployed, retired and student. ‘Other’ included, for example, freelancing and voluntary work.

^c^ The index of multiple deprivation decile is a proxy of socio-economic status^47^ derived from postcode, with lower deciles representing more socio-economic deprivation.

### Feasibility of frequent longitudinal pain self-reporting

**Figure 2.**
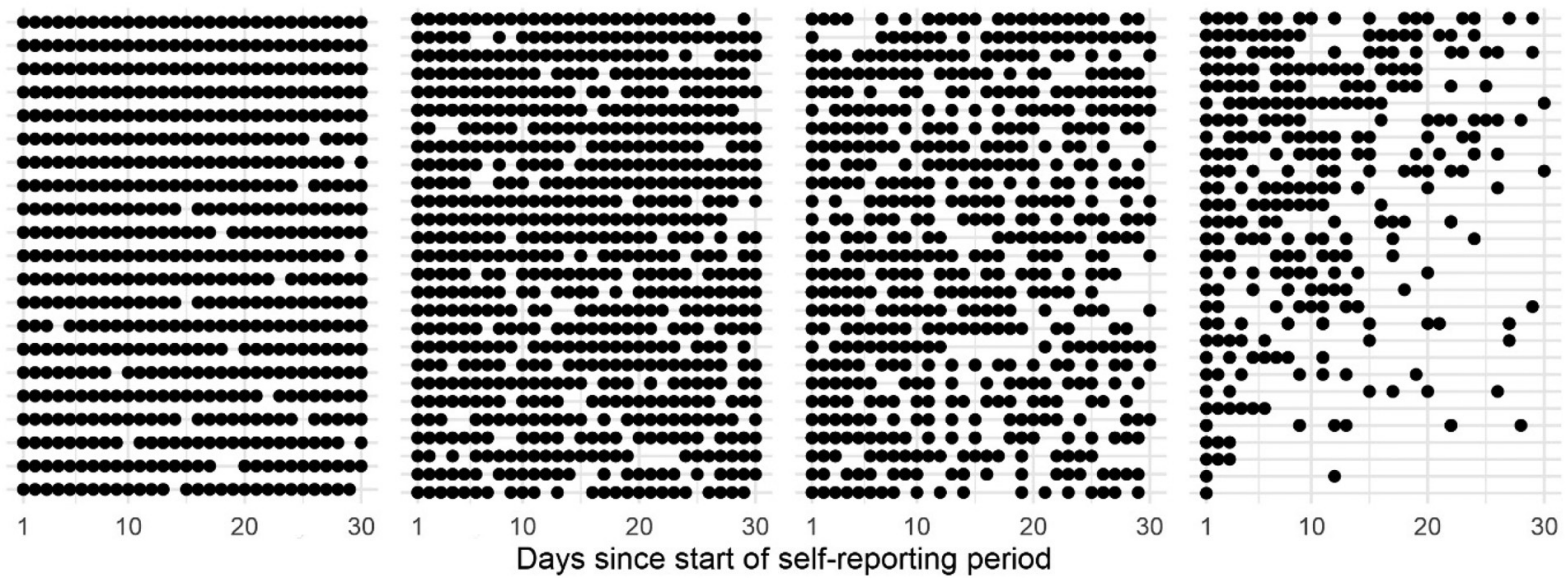
Dot plot showing daily completion for all 104 participants, ordered by level of completeness. Each row represents one participant, and each dot represents a submitted manikin report.

Participants completed a total of 2185 manikin reports across their pain self-reporting periods. The mean completion level across participants was 21 (SD, 7.7) out of 30 days. The large majority submitted manikin reports throughout the 30 days, albeit sometimes with gaps of 1–3 days without a report (see Figure 2).

**Figure 3.**
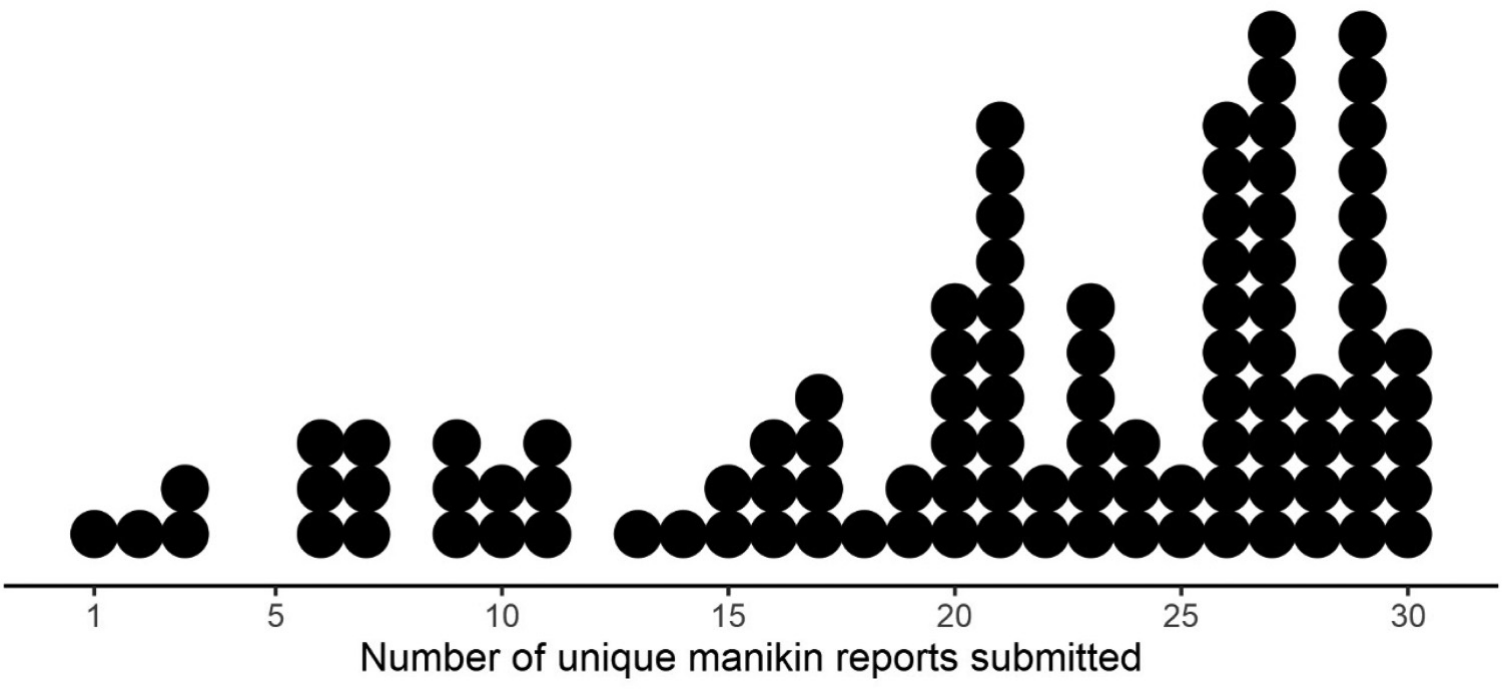
Dot histogram showing the distribution of the number of unique manikin reports submitted by each participant during their 30-day pain self-reporting period. Each dot represents one participant.

Figure 3 visualises the distribution of participants based on the number of manikin reports they submitted during their pain self-reporting period.

More than half of participants (*n* = 70; 67%) had at least one complete week with manikin reports on seven consecutive days. Eighty-nine (86%) and 44 (42%) completed two or more than two and five or more than five manikin reports in each of the four weeks, respectively.

Table 2 shows that people who were not working (OR, 1.84; 95% CI, 1.52–2.23) were more likely to complete pain self-reports than their employed counterparts. People living in the least deprived areas (OR, 0.77; 95% CI, 0.62–0.97) and from non-white ethnic backgrounds (OR, 0.45; 95% CI, 0.36–0.57) were less likely to complete pain self-reports than their more deprived and white counterparts, respectively. We did not find differences in completion levels for any of the remaining subgroups based on the participants’ gender or musculoskeletal conditions.

**Table 2.** Differences in completion levels between subgroups.

Subgroup characteristic	Categories	Number of pain reports (percentage)	Completion level mean (SD)	Adjusted OR (95% CI)^a^
Age	44 and younger	21 (20.2)	19.8 (8.9)	1.0
	45–64	59 (56.7)	20.8 (7.2)	0.91 (0.73–1.14)
	65 and older	24 (23.1)	22.5 (7.9)	1.12 (0.84–1.49)
Gender	Female	87 (83.7)	20.9 (7.9)	1.0
	Male	17 (16.3)	21.4 (6.9)	0.91 (0.71–1.17)
Ethnicity	White	89 (85.6)	21.6 (7.4)	1.0
	Non-white	15 (14.4)	17.7 (8.8)	**0.45** (**0.36–0.57)**
Employment status	Employed	45 (43.3)	19.4 (8.5)	1.0
	Not working^b^	50 (48.1)	22.6 (6.6)	**1.84** (**1.52–2.23)**
	Other^c^	9 (8.6)	19.8 (8.1)	0.96 (0.71–1.29)
Index of multiple deprivation decile^d^	1 to 3 (most deprived)	53 (51)	21 (7.3)	1.0
	4 to 6	25 (24)	21.2 (8.9)	0.98 (0.80–1.20)
	7 to 10 (least deprived)	22 (21.2)	20.9 (7.4)	**0.77** (**0.62–0.97)**
	Missing	4 (3.8)	21.2 (10.1)	1.13 (0.73–1.78)
Musculoskeletal condition	Osteoarthritis	30 (28.8)	21.6 (6.5)	1.18 (0.51–2.98)
	Rheumatoid arthritis	32 (30.8)	20.2 (8.3)	0.97 (0.43–2.39)
	Fibromyalgia	25 (24)	22 (7.7)	1.15 (0.50–2.90)
	More than one condition	17 (16.3)	22.5 (5.6)	1.19 (0.44–3.02)

OR: odds ratio; CI: confidence interval; SD: standard deviation; ref: reference group for the multivariate binomial regression model.

^a^ For each subgroup, the odd rations were adjusted for all other characteristics.

^b^ ‘White’ included all people identified as being from white ethnic backgrounds, which included mixed/multiple ethnic groups; the remaining ethnicities were recoded as ‘Non-white’.

^c^ ‘Employed’ included employed and self-employed; ‘Not working’ included unemployed, retired and student. ‘Other’ included, for example, freelancing and voluntary work.

^d^ The index of multiple deprivation decile is a proxy of socio-economic status^47^ derived from postcode, with lower deciles representing more socio-economic deprivation.

### Acceptability of longitudinal pain self-reporting

**Figure 4.**
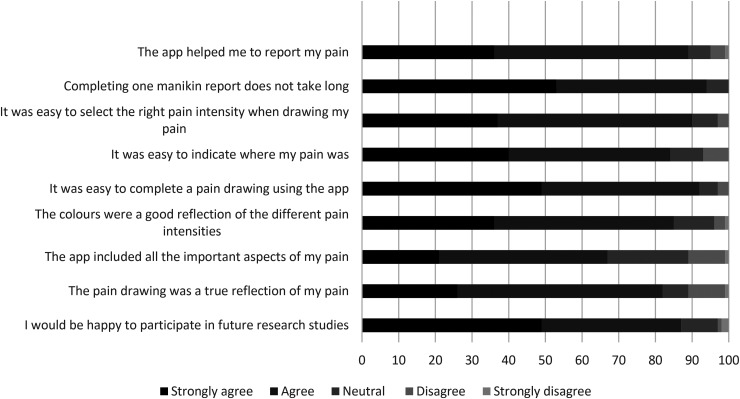
Participants’ views on the acceptability of the Manchester Digital Pain Manikin app for pain self-reporting (*n* = 96, 100%).

Of the 104 participants, 96 completed the acceptability questionnaire at the end of their pain self-reporting period. Figure 4 shows that for eight out of nine items, at least 75% participants agreed or strongly agreed that completing a pain manikin report was helpful and easy and was a good reflection of their pain. The large majority of participants (*n* = 83; 87%) also indicated they would be happy to report their pain using the Manchester Digital Pain Manikin app again for future research studies.

Reasons for not completing a daily manikin report included forgetting to complete (*n* = 40; 42%), technical issues (*n* = 13; 14%), in too much pain (*n* = 11; 11%), pain was the same as a day before (*n* = 9; 9%) or no pain to report (*n* = 4; 4%). Participants indicated that their experience of using the app would improve if they could personalise the gender (*n* = 34; 35%), body shape (*n* = 27; 28%) and skin colour (*n* = 7; 7%) of the body manikin and if the language or wording of instructions and questions would have been different (*n* = 30; 31%).

## Discussion

### Summary of main findings

We found that frequent and longitudinal pain self-reporting via a smartphone-based manikin was feasible for people living with musculoskeletal pain conditions. People who were not working were more likely, and people living in less deprived areas and from non-white ethnic backgrounds were less likely to complete pain self-reports than their employed, more deprived and white counterparts, respectively. The majority of participants found self-reporting their pain on the manikin helpful and considered the app easy to use and a good reflection of their pain. They suggested acceptability could be further enhanced by allowing personalisation of the body manikin and changing the wording or language of the instructions and questions.

### Relation to other studies

In our study, we found that people living with chronic pain found it feasible to daily self-report their pain using their own device. To our knowledge, we are the first study to evaluate the feasibility of smartphone-based digital pain manikins by assessing completion levels of daily reporting over several weeks. Other feasibility studies^35,48–51^ used manikins that were only suitable for a personal computer or laptop, collecting data at a lower frequency (e.g. once a week for 12 weeks^48^) or for a shorter period (e.g. every day for 5 days^51^). Although this may be sufficient for some purposes, studying treatment response and supporting clinical care and self-management are likely to require more frequent reporting over longer periods.^52^ Our findings suggest that digital pain manikins deployed on consumer devices can facilitate this.^53^

We also demonstrated that digital pain manikins enabled older people to self-report their pain, suggesting manikins’ potential to contribute to addressing pain under-reporting and under-treatment among elderly chronic pain populations.^54^ At the same time, however, we found that people from non-white ethnic backgrounds were less likely to self-report their pain, despite population health surveys showing that pain is more common in these groups compared to people from white ethnic backgrounds.^55^ This implies an urgent need for further efforts to enhance the cross-cultural acceptability of digital pain manikins,^28^ so that they can accurately capture these existing inequalities in pain prevalence.

Lastly, we found that the majority of our participants considered a smartphone-based manikin acceptable for pain self-reporting. This largely aligns with what previous studies found for other manikins, such as Pain-QuILT^49^ and PAIN *Report*It.^56^ However, compared to others, our manikin facilitated more precise recording of pain location and extent by allowing shading any painful body area directly on the manikin, linked to a granular underlying grid. This may have contributed to the acceptability of our manikin,^57,58^ while also enhancing its ability to support people with communicating their pain to healthcare professionals.^59–61^ Some people said they missed daily reports because they were in too much pain or because their pain was stable. This was in line with previous studies in people living with painful conditions which found that people with either flaring or well-controlled disease may not engage with pain self-reporting.^62,63^ Finally, because the manikin is available on participants’ own smartphones, they could in theory report their pain as and when they experience it. This increases our manikin's potential for capturing fluctuations in pain.^57^

### Limitations

One limitation is that our manikin app was only available for Android devices. This may have influenced the diversity of our study sample. Although some studies found very few differences between characteristics of iPhone and Android users,^64^ others found that iPhone users may be more likely to engage with health apps^65^ and more technologically literate.^66^ Consequently, the availability of our manikin app for Android only might have underestimated its feasibility. Second, the app was not available in the app store and had to be manually downloaded and installed. This may explain why out of the 131 people who consented to take part, 27 never submitted a pain report. It is likely that the manual installation process created a barrier particularly for less proficient smartphone users. This may reduce the generalisability of our findings to people with lower digital literacy levels. Third, our study was conducted without a specific intended use of the data. One can argue that engagement may have been higher if the pain reports had been integrated into clinical care, thereby opening up opportunities for the manikin data to inform participants’ disease management. For example, a study of remote monitoring of patients living with rheumatoid arthritis observed over 90% of daily symptoms being reported over 3 months with the data integrated into the electronic health record.^62^ Future studies may therefore explore the clinical utility of pain drawings to inform if and how digital pain manikin reports could be incorporated in health information systems and care pathways.

### Implications for digital pain manikin development and research

Based on our study findings, researchers should consider using a smartphone-based pain manikin as a data collection tool, in particular if they need to collect frequent, longitudinal pain data. In addition to high completion levels, it would enable automated processing of pain drawings to derive reliable and valid pain metrics, such as manikin-derived pain extent and distribution,^38^ as well as aiding diagnosis and assessment of treatment response.^21,67^

To further improve the acceptability of digital manikins for pain self-reporting, app developers should consider facilitating personalisation of the manikin appearance (e.g. of gender, body shape, skin colour) to better align with users’ individual preferences.^38,68^ For example, a more detailed, gender-specific manikin may enable women to better communicate their pain,^69^ with potential for improving their health care and outcomes.^70^ However, personalisation requirements may vary between people depending on their age, gender and ethnicity. This adds complexity of implementing such requirements into manikin software and warrants careful consideration of if and how such modifications impact the usability, reliability and validity of digital manikins as a pain measurement instrument.

Lastly, researchers should more systematically explore the health equity impact of introducing digital pain self-reporting for research, clinical care and self-management. This ensures that existing inequalities will not be widened and that opportunities to reduce these will not be missed. For example, ethnic minority groups may experience inequalities in pain prevalence and treatment outcomes because of perceived racial injustice, culturally adopted pain coping strategies and their preferences for seeking health services.^46,71^ Future studies need to actively engage with these groups to better understand their reporting preferences and requirements and how digital manikins can address these. Such studies should be guided by a structured approach to assessing the health equity impact of digital tools that involves all stakeholders, including people with lived experience.^72^ Ultimately, this will not only ensure the wider adoption of digital manikins for pain self-reporting but also help in addressing issues of health inequalities among diverse groups of people with chronic pain.

### Conclusion

People living with musculoskeletal pain conditions found it feasible and acceptable to daily self-report their pain using a smartphone-based manikin for 1 month. Future developments of digital pain manikins should focus on further optimising acceptability and ensuring equity by designing personalisable manikin apps that meet the reporting needs of a broad range of people. Ultimately, this will contribute to wider adoption of digital manikins for longitudinal pain assessments, and using them improves pain management and outcomes.

## Supplemental Material

sj-docx-1-dhj-10.1177_20552076231194544 - Supplemental material for Feasibility and acceptability to use a smartphone-based manikin for daily longitudinal self-reporting of chronic pain Click here for additional data file. Supplemental material, sj-docx-1-dhj-10.1177_20552076231194544 for Feasibility and acceptability to use a smartphone-based manikin for daily longitudinal self-reporting of chronic pain by Syed Mustafa Ali, David A Selby, Darryl Bourke, Ramiro D Bravo Santisteban, Alessandro Chiarotto, Jill Firth, Ben James, Ben Parker, William G Dixon and Sabine N van der Veer in DIGITAL HEALTH

sj-docx-2-dhj-10.1177_20552076231194544 - Supplemental material for Feasibility and acceptability to use a smartphone-based manikin for daily longitudinal self-reporting of chronic pain Click here for additional data file. Supplemental material, sj-docx-2-dhj-10.1177_20552076231194544 for Feasibility and acceptability to use a smartphone-based manikin for daily longitudinal self-reporting of chronic pain by Syed Mustafa Ali, David A Selby, Darryl Bourke, Ramiro D Bravo Santisteban, Alessandro Chiarotto, Jill Firth, Ben James, Ben Parker, William G Dixon and Sabine N van der Veer in DIGITAL HEALTH

sj-docx-3-dhj-10.1177_20552076231194544 - Supplemental material for Feasibility and acceptability to use a smartphone-based manikin for daily longitudinal self-reporting of chronic pain Click here for additional data file. Supplemental material, sj-docx-3-dhj-10.1177_20552076231194544 for Feasibility and acceptability to use a smartphone-based manikin for daily longitudinal self-reporting of chronic pain by Syed Mustafa Ali, David A Selby, Darryl Bourke, Ramiro D Bravo Santisteban, Alessandro Chiarotto, Jill Firth, Ben James, Ben Parker, William G Dixon and Sabine N van der Veer in DIGITAL HEALTH

sj-docx-4-dhj-10.1177_20552076231194544 - Supplemental material for Feasibility and acceptability to use a smartphone-based manikin for daily longitudinal self-reporting of chronic pain Click here for additional data file. Supplemental material, sj-docx-4-dhj-10.1177_20552076231194544 for Feasibility and acceptability to use a smartphone-based manikin for daily longitudinal self-reporting of chronic pain by Syed Mustafa Ali, David A Selby, Darryl Bourke, Ramiro D Bravo Santisteban, Alessandro Chiarotto, Jill Firth, Ben James, Ben Parker, William G Dixon and Sabine N van der Veer in DIGITAL HEALTH
