# Supplementary material for: Feasibility and acceptability to use a smartphone-based manikin for daily longitudinal self-reporting of chronic pain
Source: Digit Health. 2023 Aug 16;9:20552076231194544. doi: 10.1177/20552076231194544 (PMC10434844; doi:10.1177/20552076231194544)
Supplement: sj-docx-1-dhj-10.1177_20552076231194544 - Supplemental material for Feasibility and acceptability to use a smartphone-based manikin for daily longitudinal self-reporting of chronic pain [file sj-docx-1-dhj-10.1177_20552076231194544.docx]

**
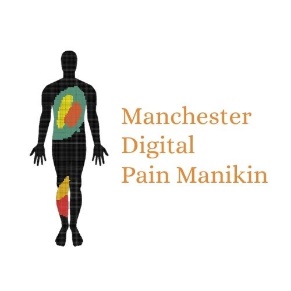
The Manchester Digital Pain Manikin Study**

**Feasibility Study**

**Baseline questionnaire**

Study ID: __________

**Instructions for completion:**

Thank you very much for taking out time to complete this survey for the Manchester Digital Pain Manikin study.

This questionnaire is to ask you 42 questions about you and your pain experience, perception and beliefs*.* There are also questions about how pain has affected your daily life and activities. Completing them will take 10-15 minutes.

You can complete this questionnaire on any digital device, but we advise a tablet, laptop or computer because they have a bigger screen. Please note that if you wish to increase the font size for ease of reading and answering, you can click on the plus sign at the top right hand corner of the questionnaire.

Please feel free to contact the research team at the University of Manchester via email on [painmanikin@manchester.ac.uk](mailto:painmanikin@manchester.ac.uk) if you need assistance. You can also use the contact details on the study flyer.

**Data collection tool:**

| **Demographics** | | |
| --- | --- | --- |
| 1 | How old are you? | 18-24  25-34  35-44  45-54  55-64  65-74  75 or older |
| 2 | What is your gender? | Male  Female  Non-binary/ third gender  Prefer not to say  Prefer to self-describe |
| 3 | What is the highest level of education you have completed? | Nil  Primary school  Secondary school  Further education (e.g., A-level, BTEC)  College or university  Post-graduate degree  Prefer not to say |
| 4 | Please enter your postcode | ______________________________ |
| 5 | What is your ethnicity? | White (British and others)  Asian or Asian British - Pakistani  Asian or Asian British – Indian  Asian or Asian British – Bangladeshi  Asian or Asian British – Chinese  Asian or Asian British – Others  Black or Black British - African  Black or Black British - Caribbean  Black or Black British - Others  Mixed or multiple ethnic group (White and others)  Other  Prefer not to answer |
| 6 | What is your employment status? | Employed  Self-employed  Unemployed  Retired  Student  Other (please specify) |
| 7 | Is English your native language? | Yes  No |
| 8 | How easy is it for you to understand written English? | Very easy  Somewhat easy  Neither easy nor difficult  Somewhat difficult  Very difficult |

| **Pain experience** | | |
| --- | --- | --- |
| 9 | How long you have been experiencing pain? | Less than a year  1-3 years  4-10  More than 10 years |
| 10 | With whom do you talk about your pain? (select all options that apply) | Family members  Friends  Health care professional (e.g. your GP, hospital doctor)  Other (please specify) |
| 11 | How much pain did you have in the last 7 days?  (0 is no pain and 10 is worst imaginable pain) | 0  1  2  3  4  5  6  7  8  9  10 |
| 12 | If 0 is “no pain” and 10 is “worst imaginable pain”, then I consider my pain unbearable when it reaches a score of___; | 0  1  2  3  4  5  6  7  8  9  10 |

| **Pain reporting behaviour and preference**  Indicate for each of the following situations if you would normally report your pain to a healthcare professional (e.g. your GP, a hospital doctor): | | |
| --- | --- | --- |
| 13  14  15  16  17  18  19 | As soon as I am in pain, no matter how severe it is  As soon as my pain becomes unbearable  When my pain is persistent but bearable  When my pain is persistent and unbearable  When pain intensity is persistently higher than usual  When my pain persists longer than usual, no matter how severe it is  As soon as my pain stops me from ……….. (e.g. sleeping, going for my daily walk, etc) | [Yes and No options for all statements]  Yes No  Yes No  Yes No  Yes No  Yes No  Yes No  _____________________ |

| **Pain perception and beliefs**  Indicate to what extent you agree with each of the following statements | | |
| --- | --- | --- |
| 20 | My pain varies in intensity but is always with me. | Strongly agree  Agree  Disagree  Strongly Disagree |
| 21 | I don't know enough about my pain. | Strongly agree  Agree  Disagree  Strongly Disagree |
| 22 | If I am in pain it is my own fault. | Strongly agree  Agree  Disagree  Strongly Disagree |

**Global Pain Scale**

*Your pain*

**For each question, please indicate your level of pain by selecting a number from 0 to 10**

| My current pain is | No pain 0 1 2 3 4 5 6 7 8 9 10 Extreme pain |
| --- | --- |
| During the past week, the best my pain has been is | No pain 0 1 2 3 4 5 6 7 8 9 10 Extreme pain |
| During the past week, the worst my pain has been is | No pain 0 1 2 3 4 5 6 7 8 9 10 Extreme pain |
| During the past week, my average pain has been | No pain 0 1 2 3 4 5 6 7 8 9 10 Extreme pain |
| During the past 3 months, my average pain has been | No pain 0 1 2 3 4 5 6 7 8 9 10 Extreme pain |

*Your feelings*

**During the past week I have felt**

| Afraid | Strongly disagree 0 1 2 3 4 5 6 7 8 9 10 Strongly agree |
| --- | --- |
| Depressed | Strongly disagree 0 1 2 3 4 5 6 7 8 9 10 Strongly agree |
| Tired | Strongly disagree 0 1 2 3 4 5 6 7 8 9 10 Strongly agree |
| Anxious | Strongly disagree 0 1 2 3 4 5 6 7 8 9 10 Strongly agree |
| Stressed | Strongly disagree 0 1 2 3 4 5 6 7 8 9 10 Strongly agree |

*Your clinical outcomes*

**During the past week**:

| I had trouble sleeping | Strongly disagree 0 1 2 3 4 5 6 7 8 9 10 Strongly agree |
| --- | --- |
| I had trouble feeling comfortable | Strongly disagree 0 1 2 3 4 5 6 7 8 9 10 Strongly agree |
| I was less independent | Strongly disagree 0 1 2 3 4 5 6 7 8 9 10 Strongly agree |
| I was unable to work (or perform normal tasks) | Strongly disagree 0 1 2 3 4 5 6 7 8 9 10 Strongly agree |
| I needed to take more medication | Strongly disagree 0 1 2 3 4 5 6 7 8 9 10 Strongly agree |

*Your activities*

**During the past week I was NOT able to**:

| Go to the store | Strongly disagree 0 1 2 3 4 5 6 7 8 9 10 Strongly agree |
| --- | --- |
| Do chores in my home | Strongly disagree 0 1 2 3 4 5 6 7 8 9 10 Strongly agree |
| Enjoy my friends and family | Strongly disagree 0 1 2 3 4 5 6 7 8 9 10 Strongly agree |
| Exercise (including walking) | Strongly disagree 0 1 2 3 4 5 6 7 8 9 10 Strongly agree |
| Participate in my favorite hobbies | Strongly disagree 0 1 2 3 4 5 6 7 8 9 10 Strongly agree |
