# Supplementary material for: Feasibility and acceptability to use a smartphone-based manikin for daily longitudinal self-reporting of chronic pain
Source: Digit Health. 2023 Aug 16;9:20552076231194544. doi: 10.1177/20552076231194544 (PMC10434844; doi:10.1177/20552076231194544)

**The Manchester Digital Pain Manikin Study**

**
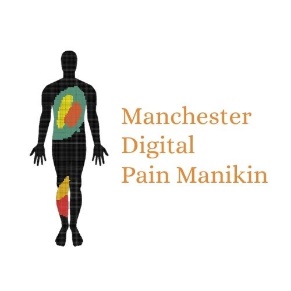
**

**HOW TO GUIDE**


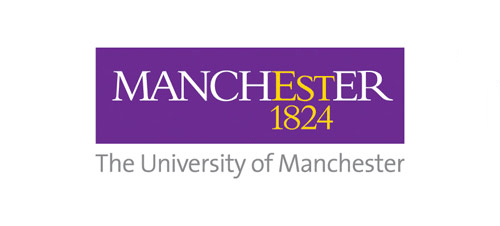


# Content

| [**A**](#_How_to_download,) | [**B**](#_B._How_to) |
| --- | --- |
| 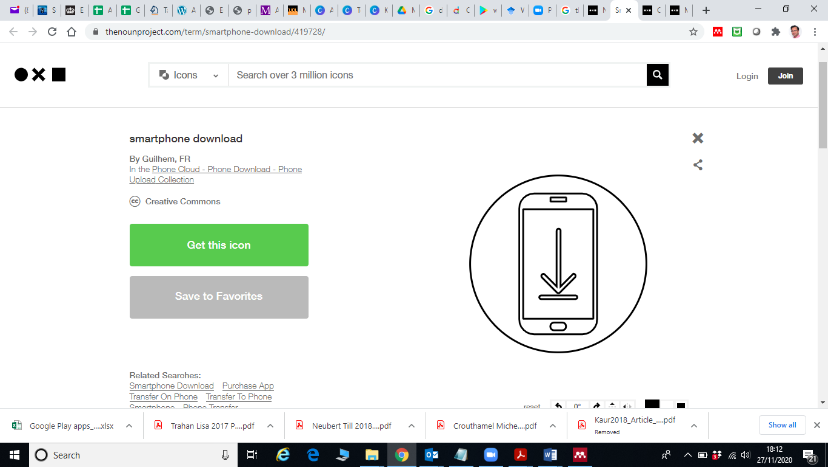  How to download and install the app on your smartphone | 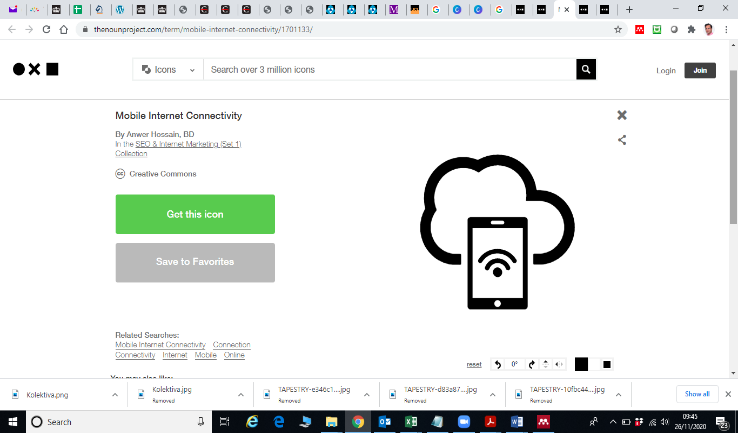  How to check if your phone is connected to the internet |
| [**C**](#_C._How_to) | [**D**](#_How_to_get) |
| **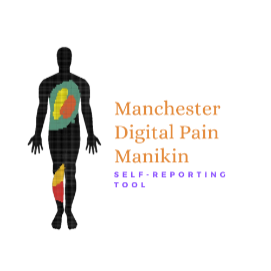**  How to self-report your pain | 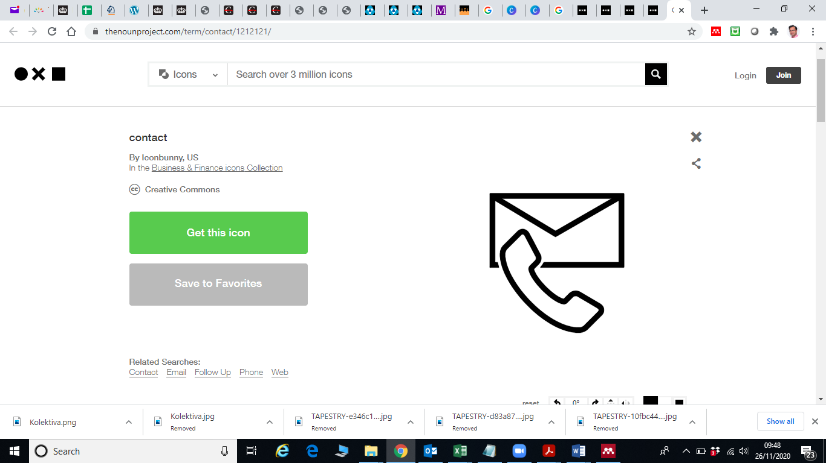  How to get in touch if you need help |

If you have any questions or concerns about the information in this guide, go to section D for more information on, [“How to get in touch if you need help”.](#_How_to_get)


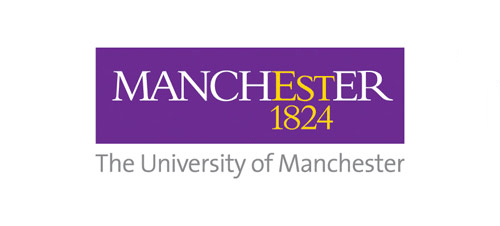


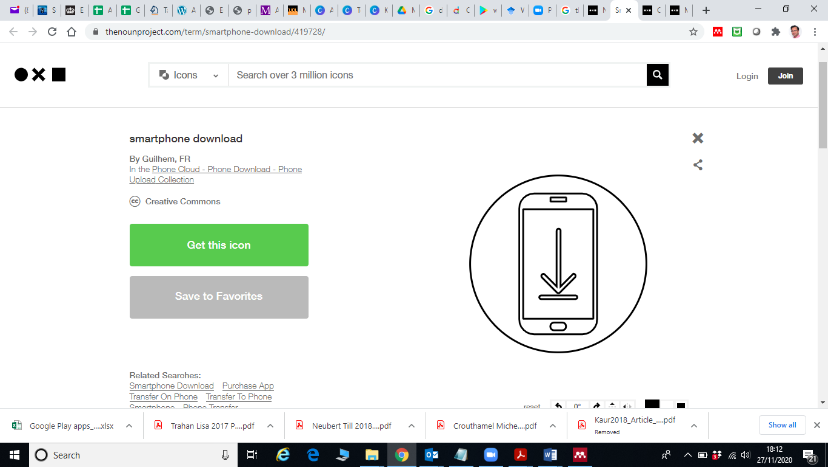


# A. How to download and install the app on your smartphone

Please follow the steps below to download and install the Manchester Digital Pain Manikin app on your smartphone.

Please note the procedure may vary based on the model of your phone and/or Android version of your phone. Therefore, we are offering optional on-boarding support via Zoom, should you need help with downloading and installing the app.

1. After you have completed the baseline questionnaire, you will receive a link via email on the address you provided (see Figure A1). You can download and install the app by clicking the link. This will only work if you have an Android phone (that is, any phone that is not an iPhone).
2. Select the personal Gmail account of your choice, if prompted (see figure A2)
3. Open with ‘Package installer’, as suggested by your phone. Just tap on the ‘Package installer’ option (see figure A3).

| 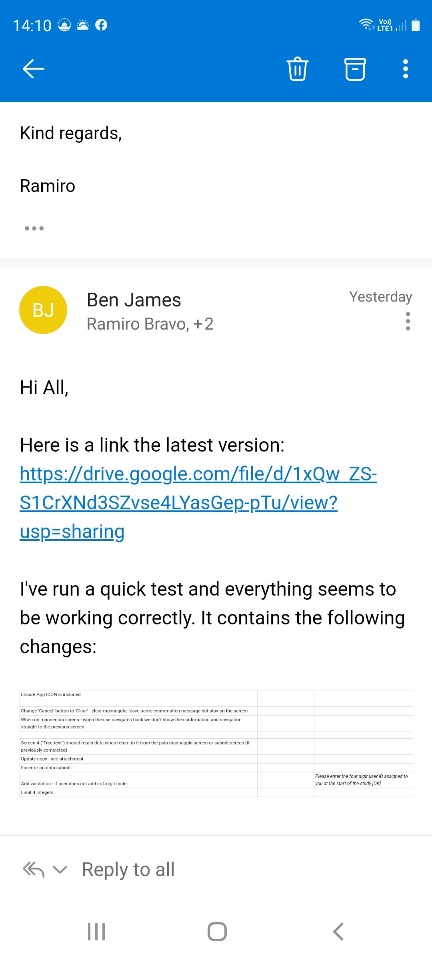 | 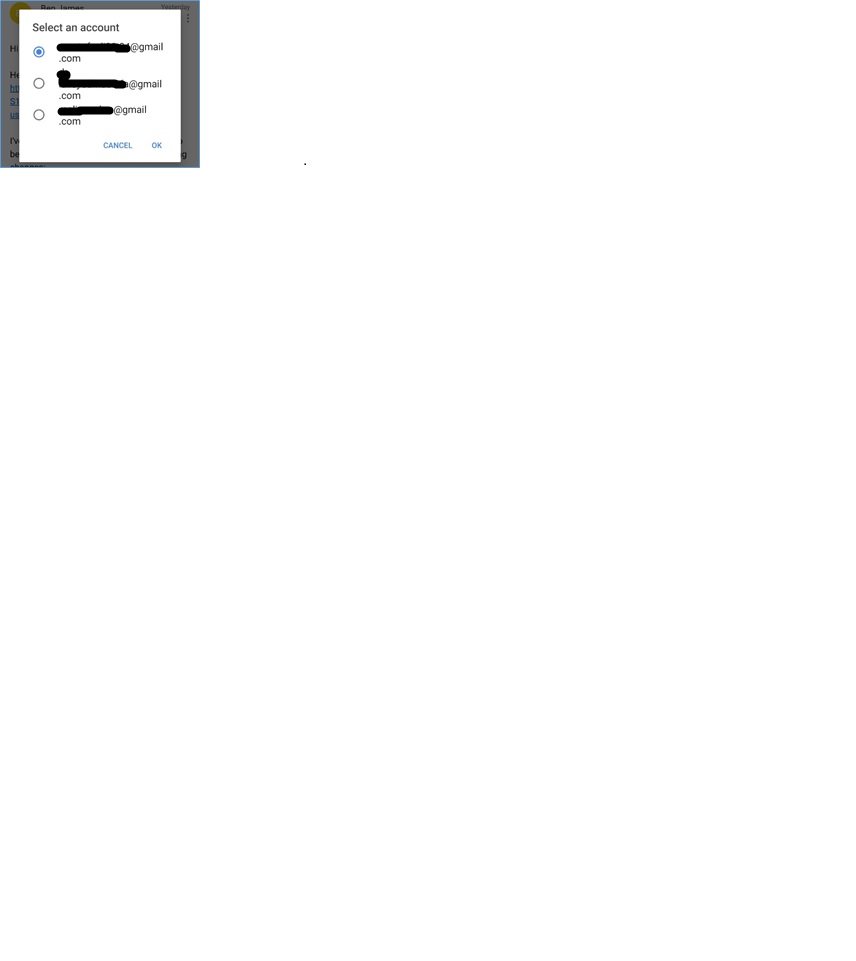 | 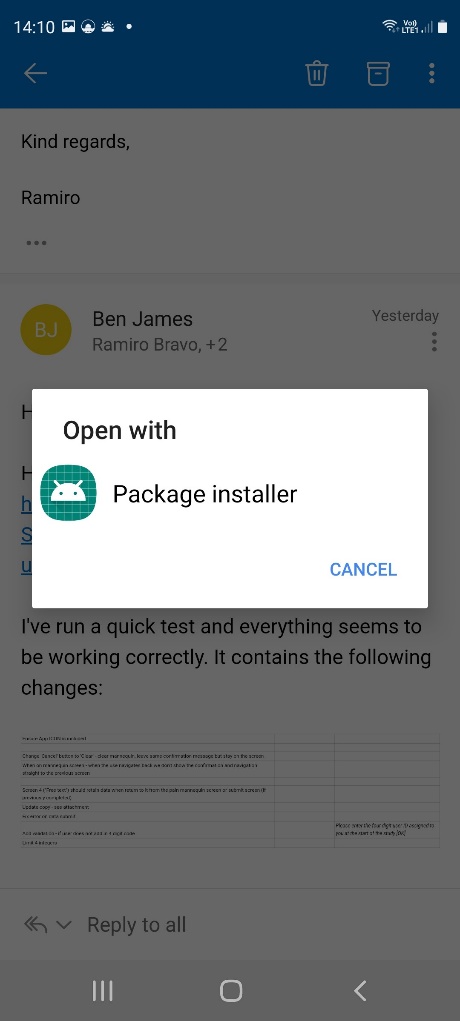 |
| --- | --- | --- |
| A1. Click app download link | A2. Select your Gmail account | A3. Open with ‘Package installer’ |

1. Tap ‘Install’ to start installation
2. Installing normally takes less than a minute to complete
3. Once the app is installed, you can tap ‘Open’ to start using the app

| 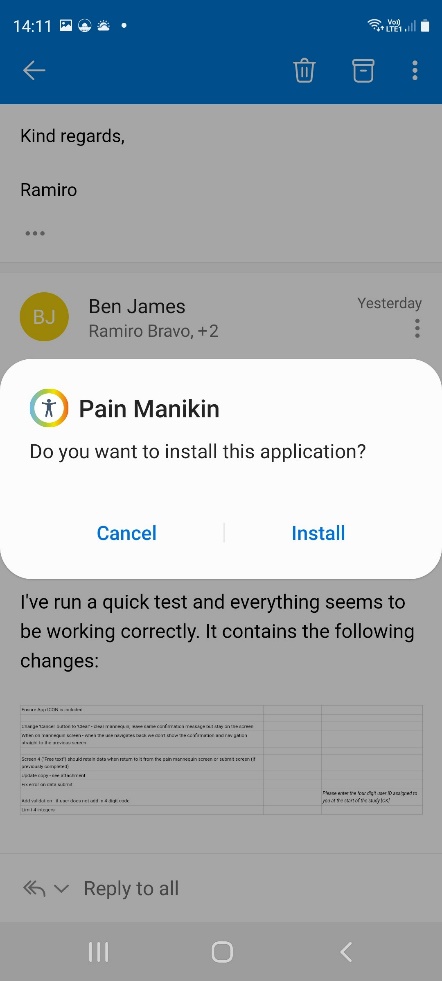 | 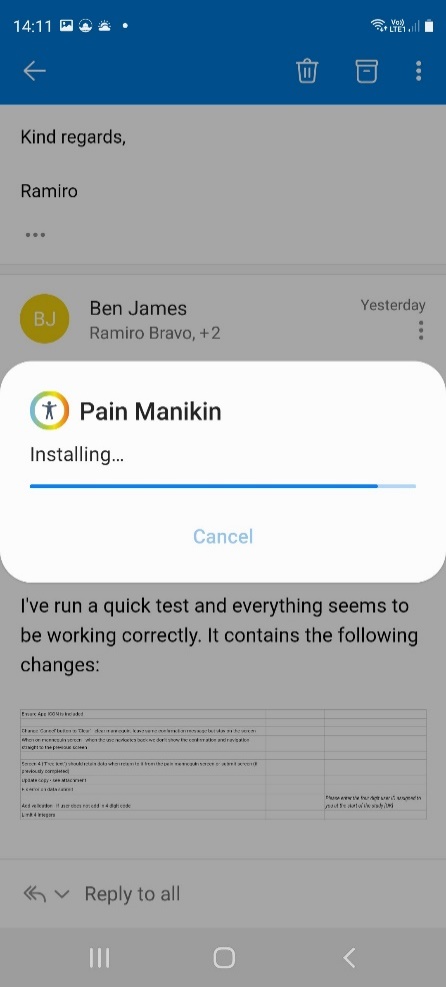 | 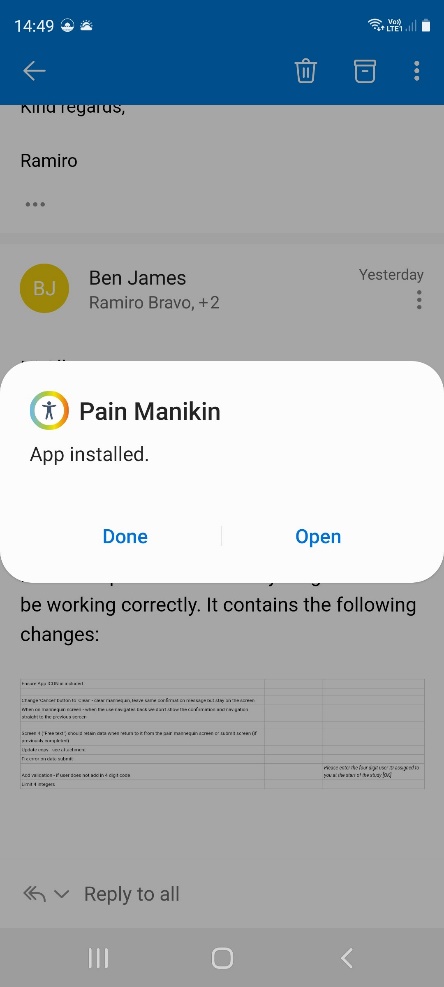 |
| --- | --- | --- |
| A4. Begin installation | A5. Installation in progress | A6. Installation completed |

If you completed steps 4-6 successfully, you can skip steps 7-9 below. Depending on your phone’s settings, the download and installation procedure might be slightly different from the above description. You might have to take additional steps 7-9 outlined below to start installation.

7. As the app is shared via Google Drive, you will have to manually change settings to start installation. Press ‘Settings’ (see figure A7).

8. Move the slider (circled in red in figure A8) to the right to allow the download from Google Drive.

9. Now complete steps 4-6 as described above.

| 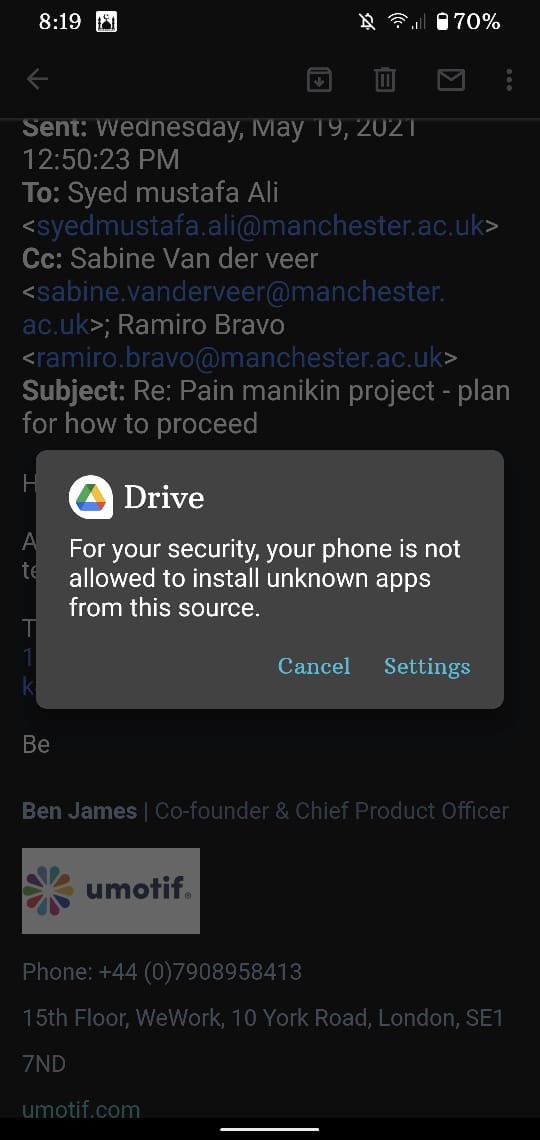 | 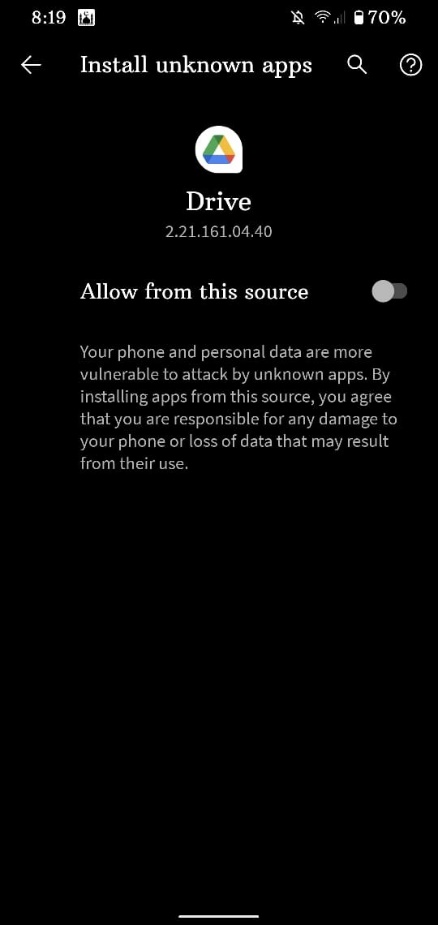 | 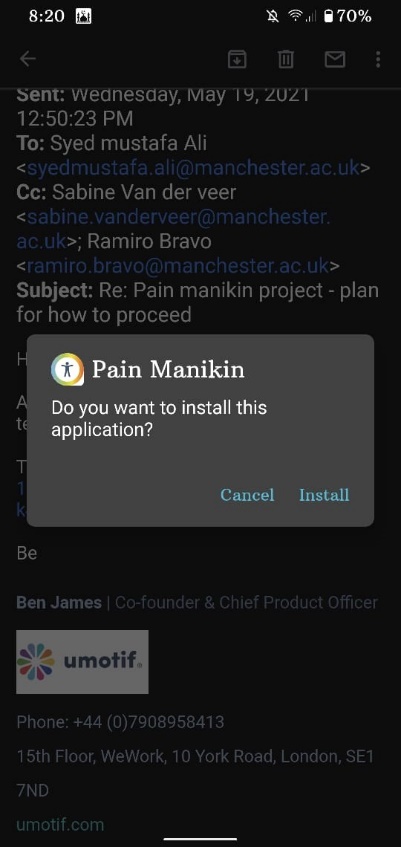 |
| --- | --- | --- |
| A7. Change the settings | A8. Allow installation from Google drive source | A9. Begin installation |


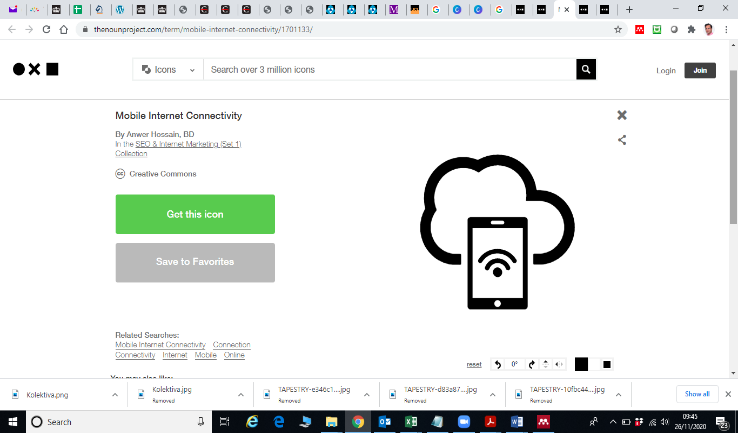


# B. How to check if your phone is connected to the internet

Every time before you use the app to report your pain, please check that your phone is connected to the internet. Otherwise, you will not be able to submit your manikin report.

1. To quickly check if you are connected to internet or not, check for one of the following two icons. You can find them at the top of your phone screen, near the battery sign (see figure B1 & B2):


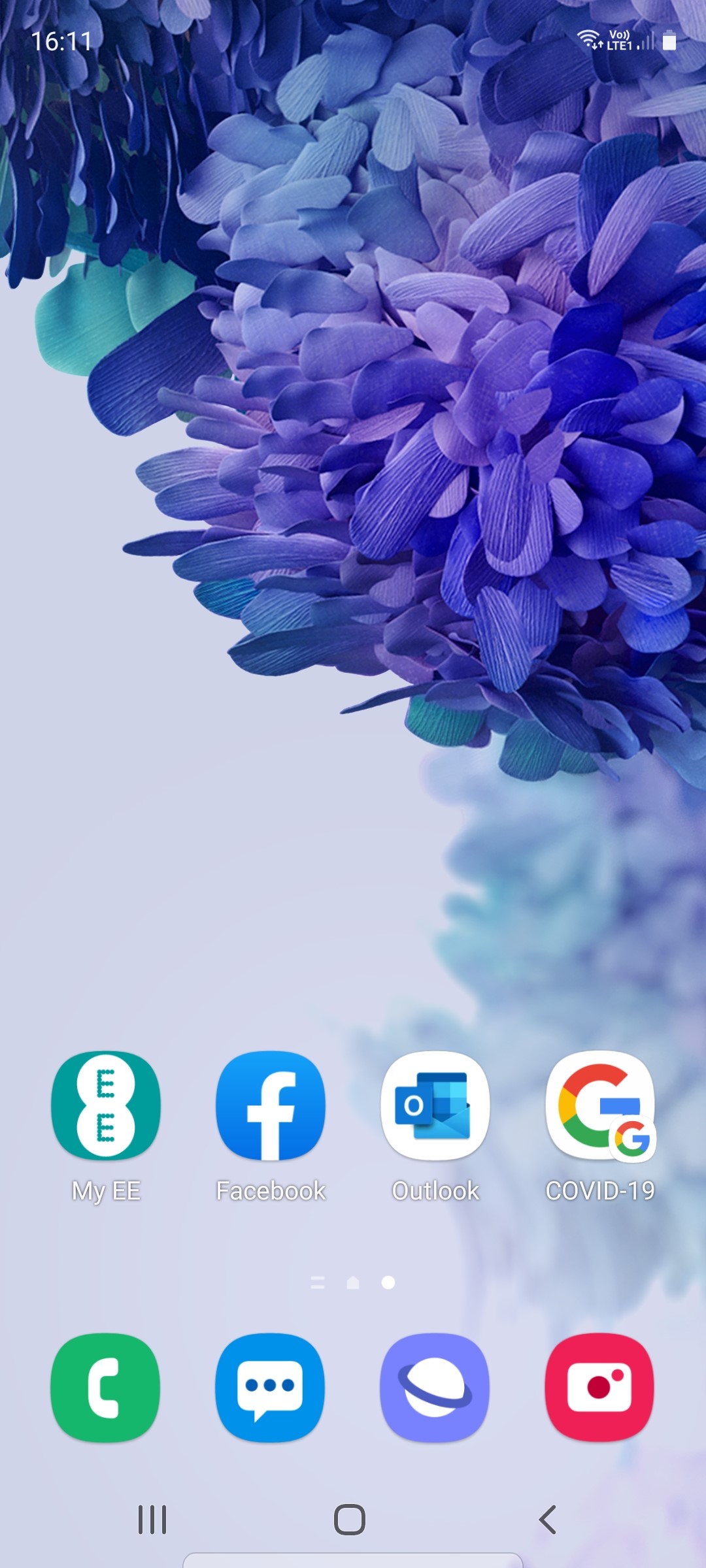

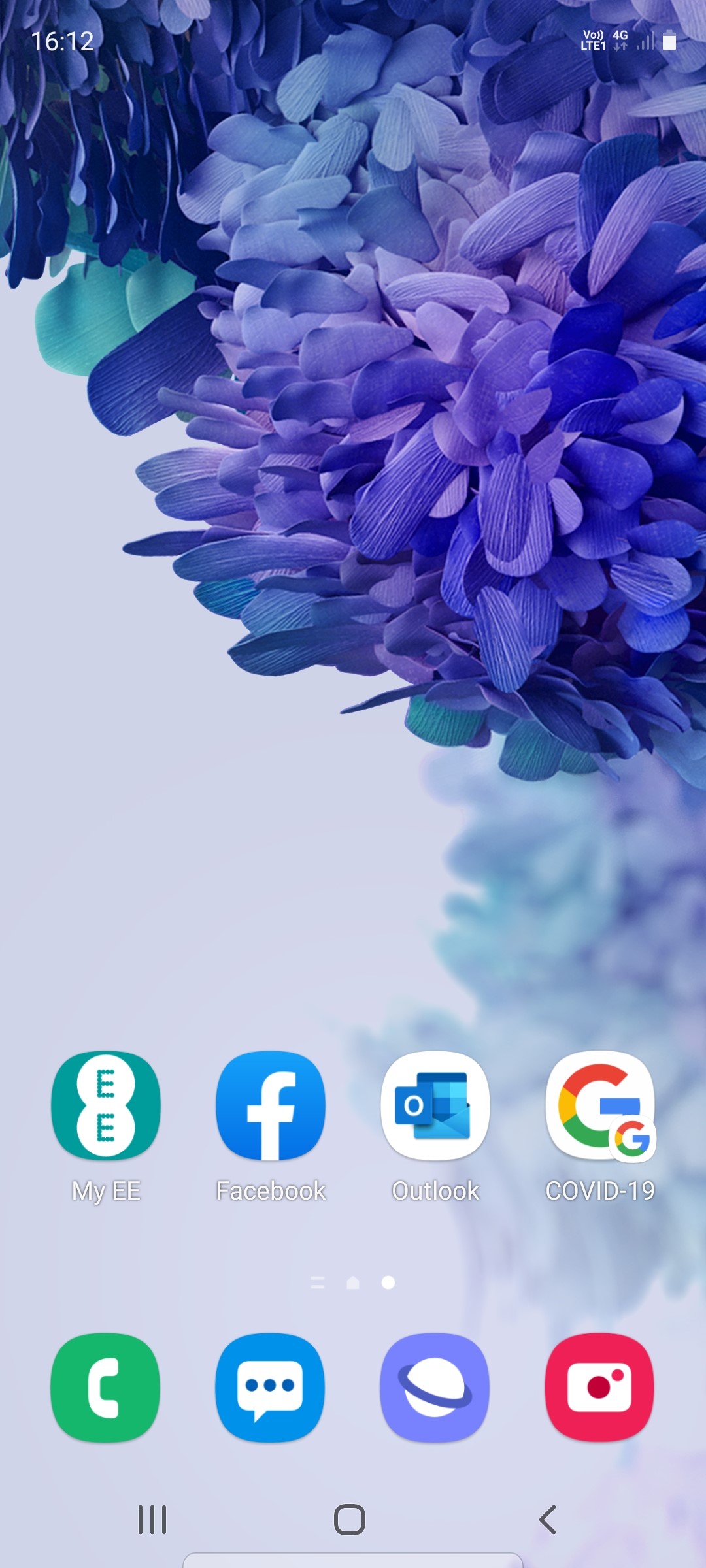


B1. An inverted triangle with double arrows means you are connected to the internet via Wi-Fi

B2. 4G or 3G with double arrows means you are connected to the internet via mobile data

If you do not see either of the above signs, there are two ways to connect to the internet:

1. Go into ‘Settings’ and look for ‘Connections’. Within ‘Connections’ find ‘Wi-Fi’ and turn it on by moving the small circle to the other end (see figures B3);
2. Swipe down when you are on your phone’s home screen, and tap ‘Wi-Fi’ or ‘Mobile data’ (see figure B4).

| 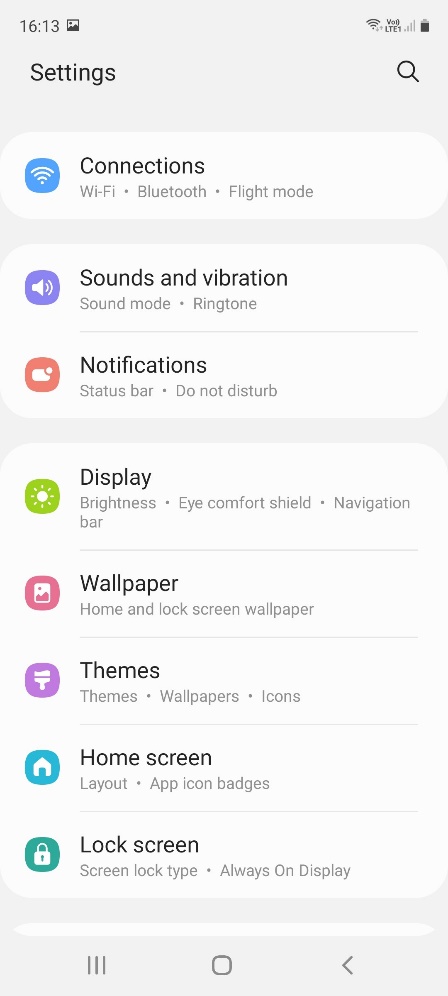 | 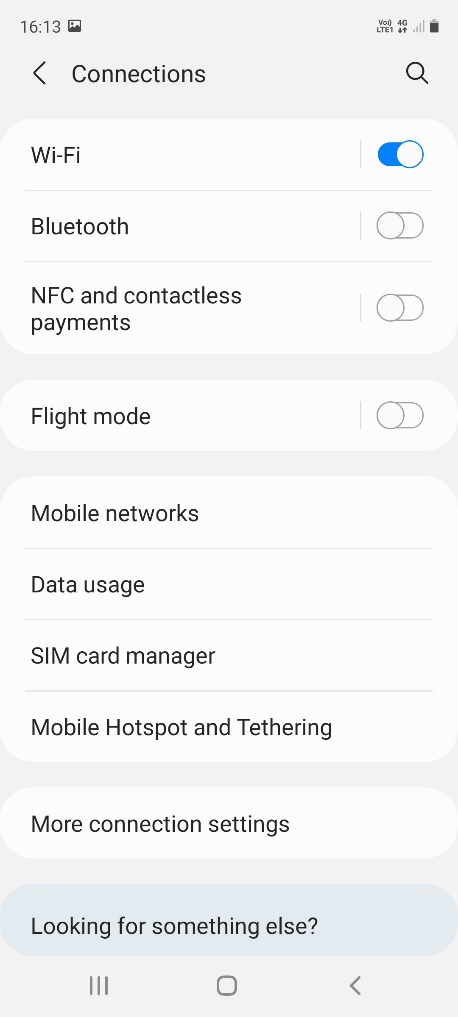 | 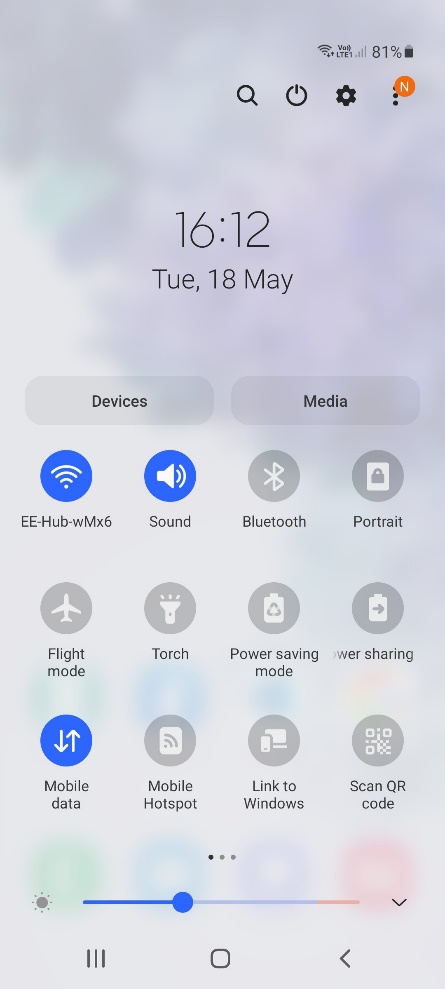 |
| --- | --- | --- |
| B3. Go into connection settings | B3. Turn on Wi-Fi | B4. Shortcut to enable connectivity |

**
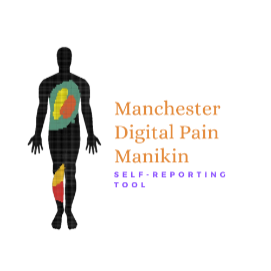
**

# C. How to self-report your pain

Before you start self-reporting your pain in the app, we recommend you activate a screen lock on your phone to ensure that nobody else can report and submit pain reports without your permission.

Once the app is installed, **you will receive a reminder every day at 8pm** to complete and submit your manikin report (see figure C1). However, you can complete and submit your daily manikin report at any time of the day, whenever it is convenient and relevant for you. If you do this before 8pm on a certain day, you can ignore the reminder for that day.

| 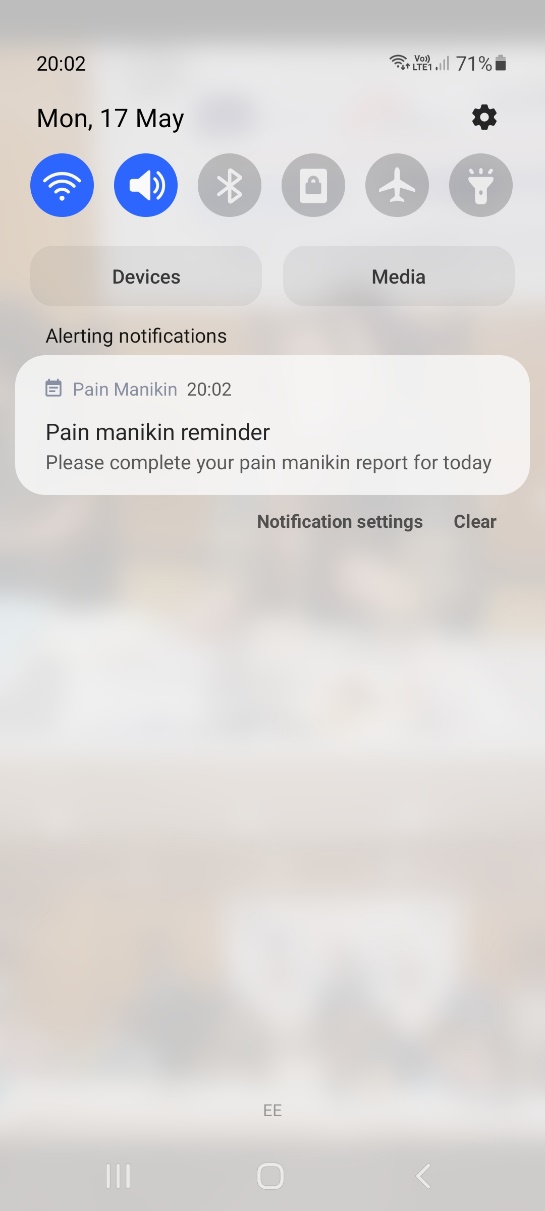 |
| --- |
| C1. Daily pain manikin reminder |

The app has five screens in total. In one pain reporting session, you can move back and forth between the screens without losing information. **Before you start your daily report, make you are connected to internet (see section B): you cannot save your report and submit it later**.

**Screen 1** provides information on what is included in a daily pain manikin report and **instructions** for how to complete a pain drawing (see figure C2).

On **screen 2,** you can indicate **how much pain you had overall** on that day. Use the slider to choose any number between 0 (‘No pain’) and 10 (‘Worst pain imaginable’) that best represents your overall pain for that day (figure C3).

| 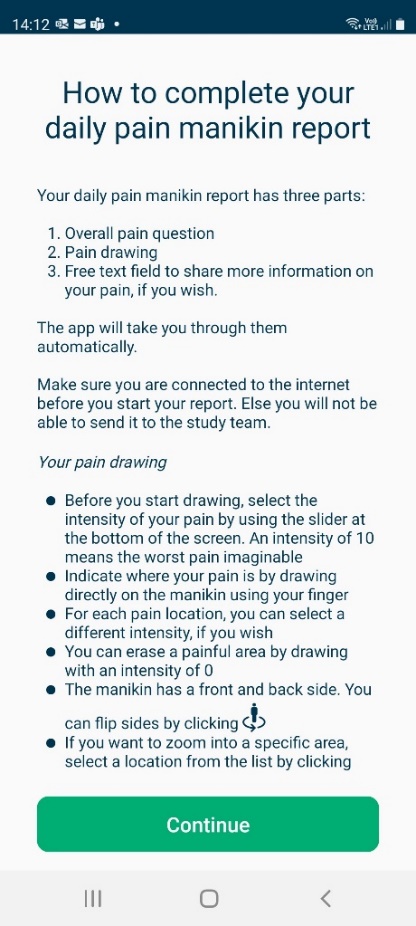 | 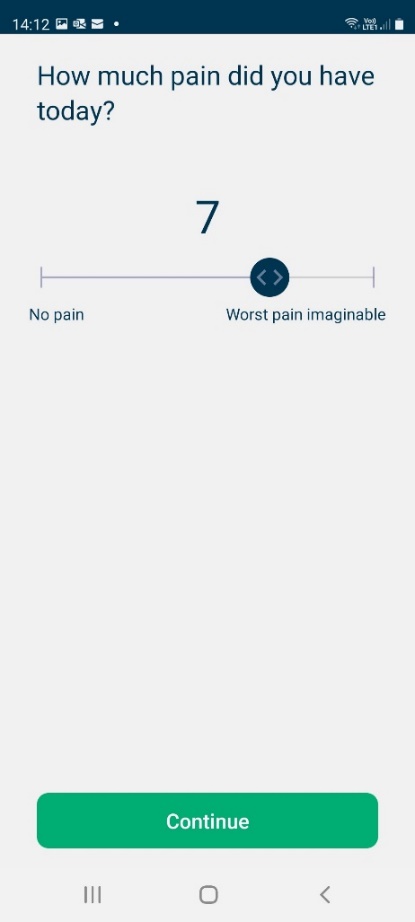 |
| --- | --- |
| C2. Screen 1: Instructions | C3. Response to overall pain question (example) |

On **screen 3**, you can complete your **pain drawing** by drawing directly on the manikin using your finger. The manikin has a front (Figure C4) and a back (Figure C5) view, as well as zoomed in views for specific body parts (Figure C6).

| 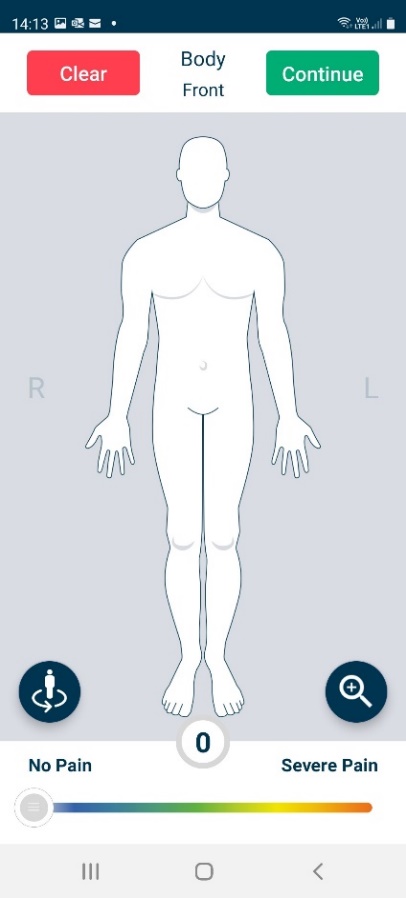 | 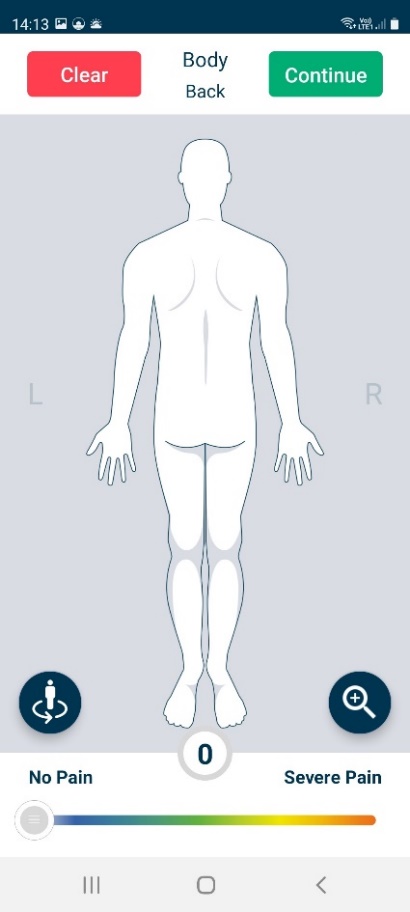 | 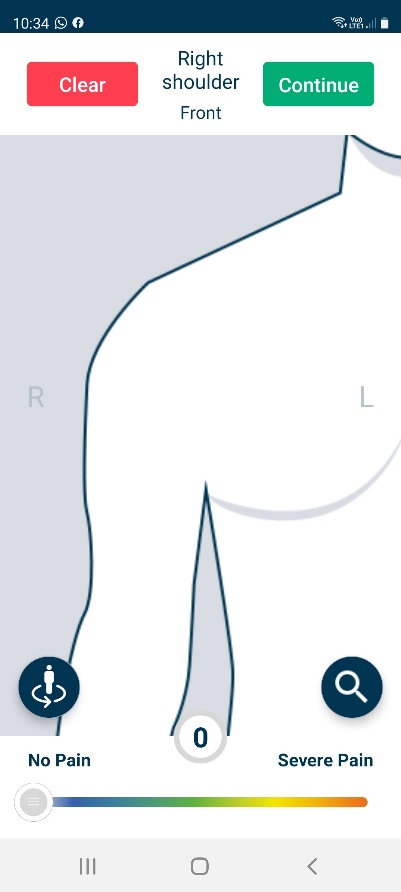 |
| --- | --- | --- |
| C4. Front view of the manikin | C5. Back view of the manikin | C6. Zoomed in view of right shoulder |

This table explains **what the icons, buttons and letters on screen 3 mean**:

| 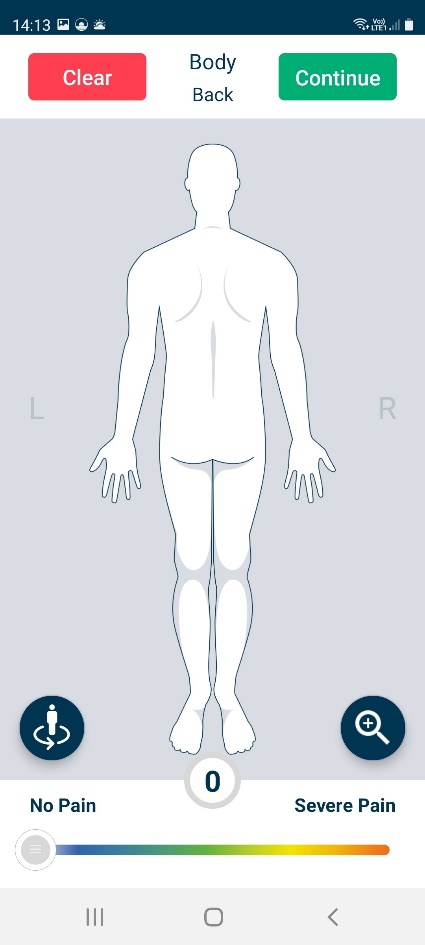 | Flip between the front and the back of the manikin |
| --- | --- |
| 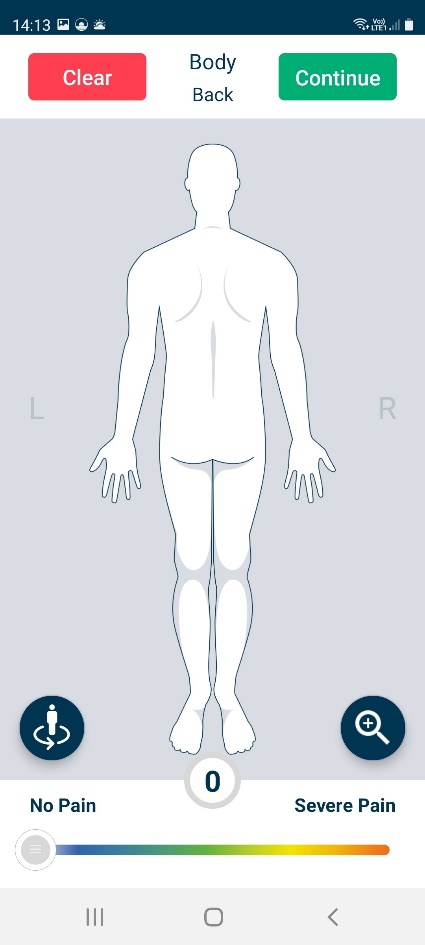 | Select a zoomed in view of a specific body part. When you tap this icon, you will see a list (see Figure C7). Here you can choose what body part you want to zoom into.  If you want to return to the full body view, tap this icon and select ‘Body’ from the list. |
| R / L | Indicate what are the Right and Left side of the body. These letters will swap if you flip between the front and back of the manikin |
| 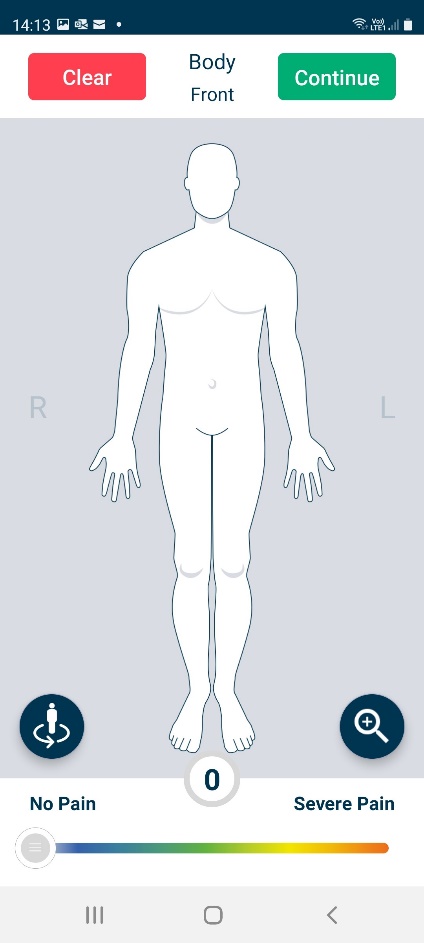 | The text above the manikin tells you what body part you are viewing and whether it is the front or back. This text will change depending on what view you select |
| 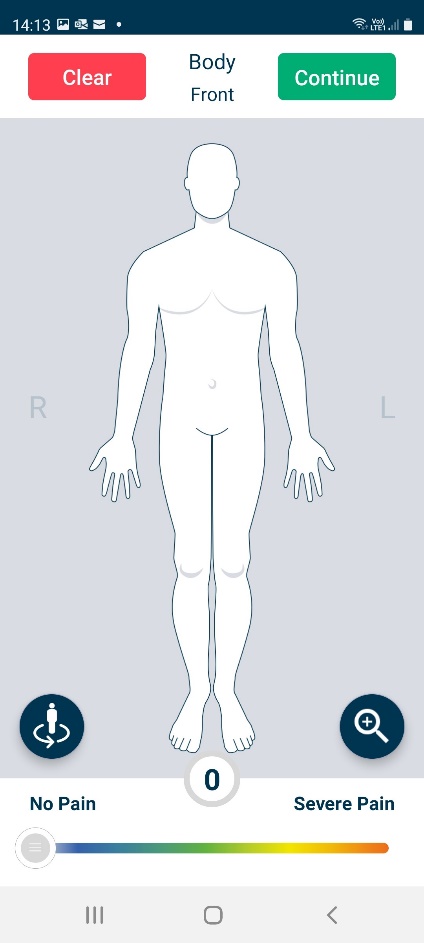 | The red ‘clear’ button resets your manikin by erasing all the areas you shaded. You can use this button if you are unhappy with your drawing and want to start over again |
| 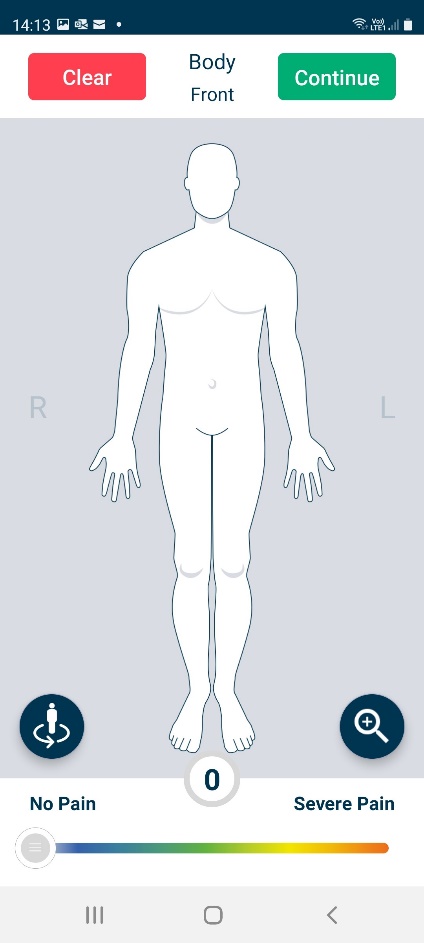 | The green ‘continue’ button takes you to screen 4 once you have completed your drawing. |

| 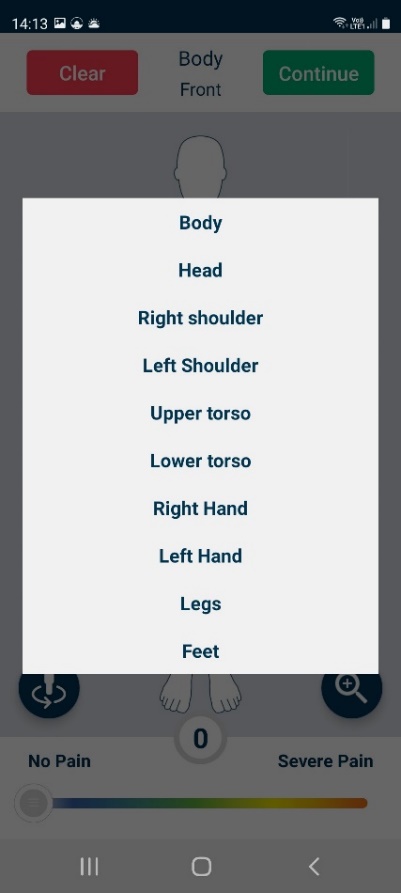 |
| --- |
| C7. List of pre-specified enlarged body parts |

Using the buttons and icons described in the table, **you can draw your pain as follows**:

1. Select the correct view (front-back; whole body or zoomed in body area)
2. Select the intensity of your pain by using the slider at the bottom of the screen. An intensity of 10 means the worst pain imaginable
3. Indicate where your pain is by drawing directly on the manikin using your finger
4. For each painful location, you can select a different intensity, if you wish
5. You can erase a painful area by drawing with an intensity of 0

Once you have completed your pain drawing as accurately as possible, tap the ‘Continue’ button to go to the next screen.

**If you have a pain-free day**, you can leave the pain drawing blank for that day.

| 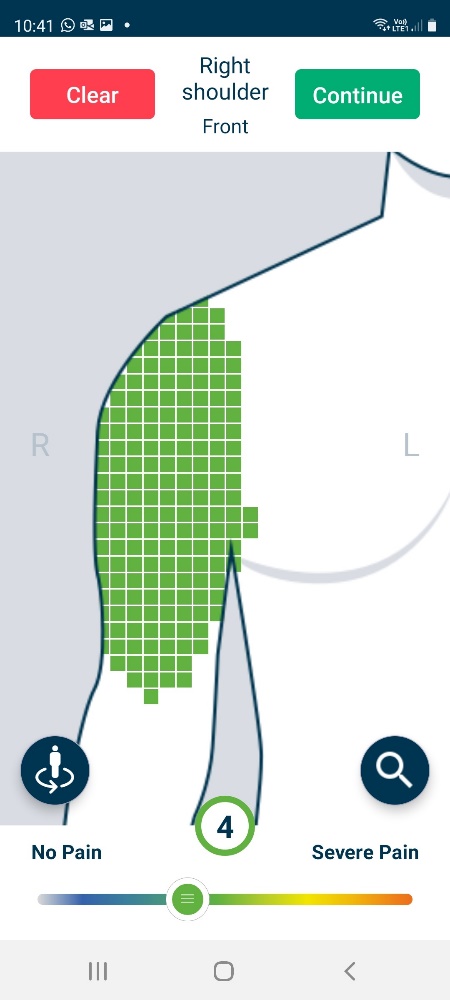 | 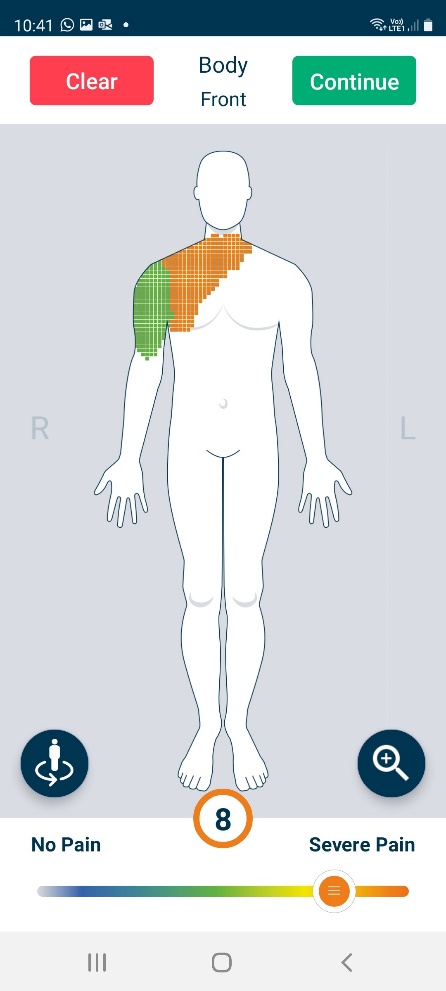 | 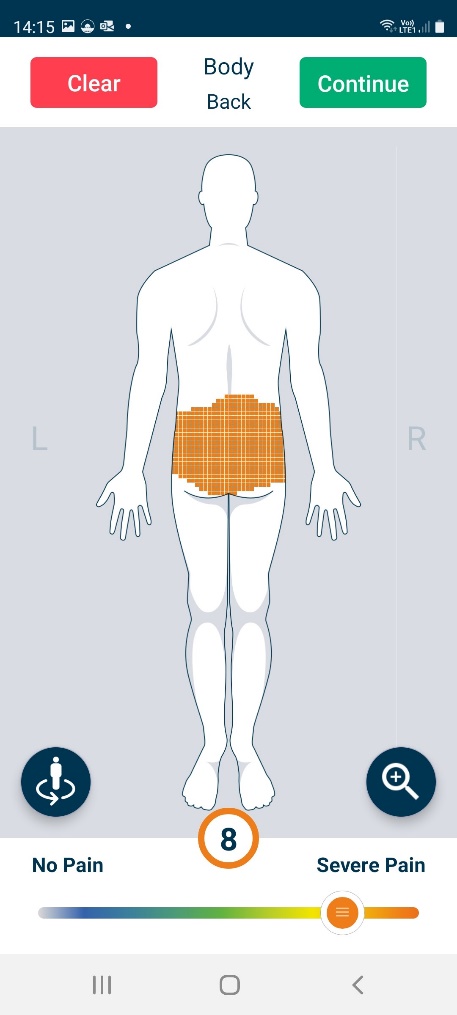 |
| --- | --- | --- |
| C7. Draw pain with intensity of 4 in zoomed in front view of shoulder | C7. Draw pain with intensity of 8 in front view of body | C7. Draw pain with intensity of 8 in back view of body |


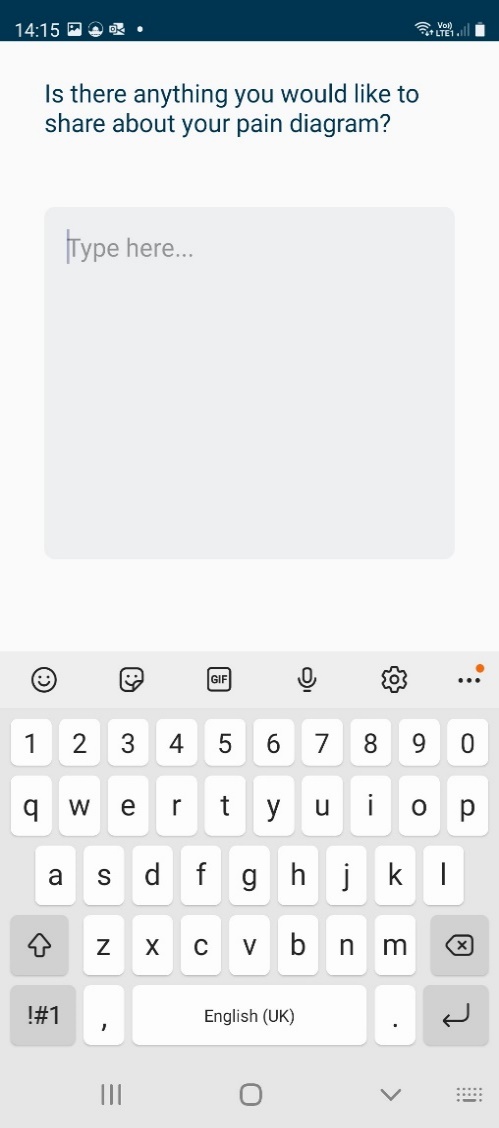
**Screen 4** gives you the option to **provide additional information about your pain** or pain drawing, if you want to. For example, to describe your pain for that day in your own words, how it made you feel, what impact it had on your daily life, or what you think made it worse or better than the day(s) before. To start typing, tap in the grey area for the keyboard to appear (see figure C8). Once you are ready to move on to the next screen, tap for the keyboard to disappear (see figure C9). Then tap ‘Continue’.

**If you have a pain free day**, please type ‘No pain today’ in this field.

| 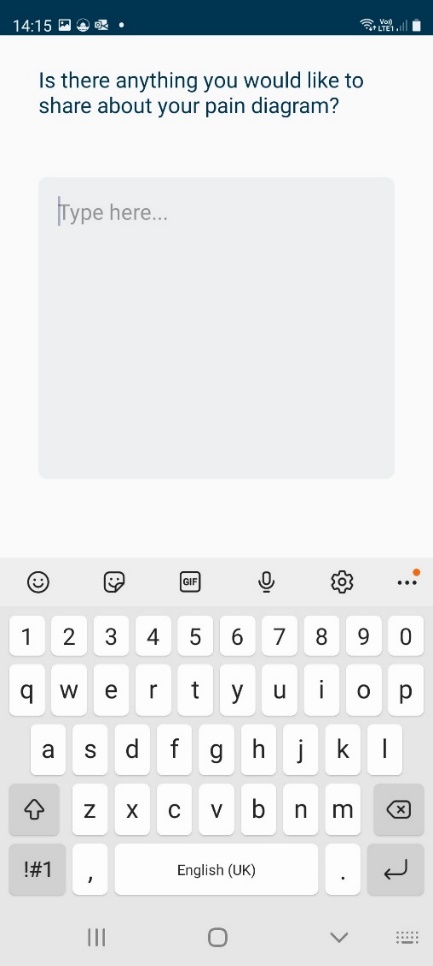 | 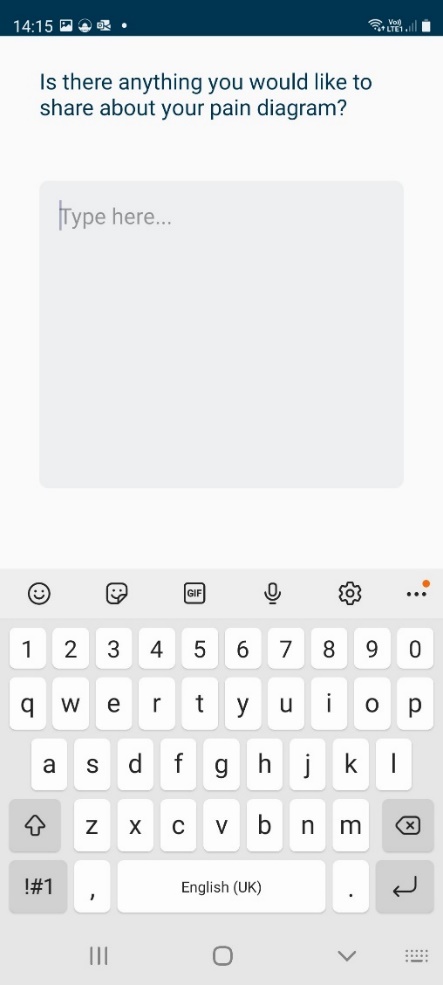 |
| --- | --- |
| C8. Tap in the grey box for the keyboard to appear | C9. Tap on downward arrow head for the keyboard to disappear |

**Screen 5** is the last screen. Here you can **submit your manikin report** (figure C10).

When submitting your first manikin report, you will need to enter **your user ID**. This is a four digit number that is unique to you, so **it is important to enter it correctly**. You can find it in the email you received from the study team when sending you the link for downloading the app or during the online on-boarding session ([see section A](#_How_to_download,)). If you cannot find your user ID, please contact the study team ([see section D](#_How_to_get)) before submitting any reports.

Once you have entered the user ID once, the app will remember it. So, you do not have to re-enter it again.

| 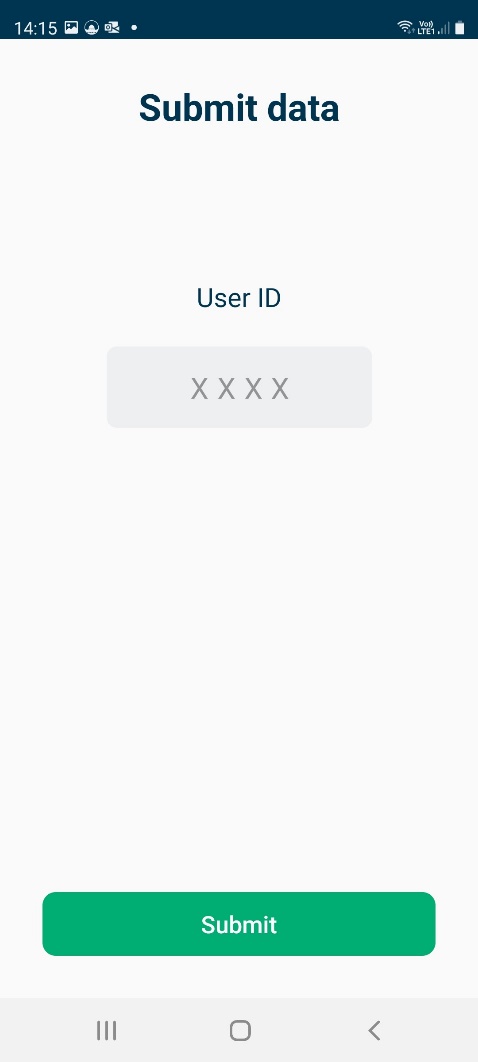 | 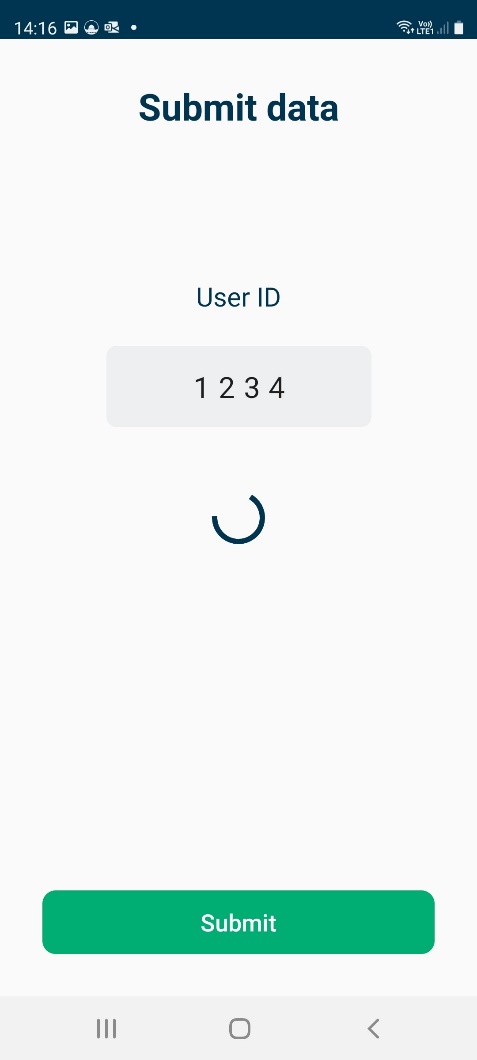 |
| --- | --- |
| C10. Submission screen | C11. Enter four digit unique user ID |

Confirm that your phone is still connected to the internet (section B) and tap ‘Submit’. You will see a message that the report has been submitted successfully (Figure C12).

You will get an error message if:

- Your user ID is incorrect (Figure C13) – please check the number you received from the study team and correct
- You do not have internet connection (Figure C14) – please connect to the internet and submit your report again.

| 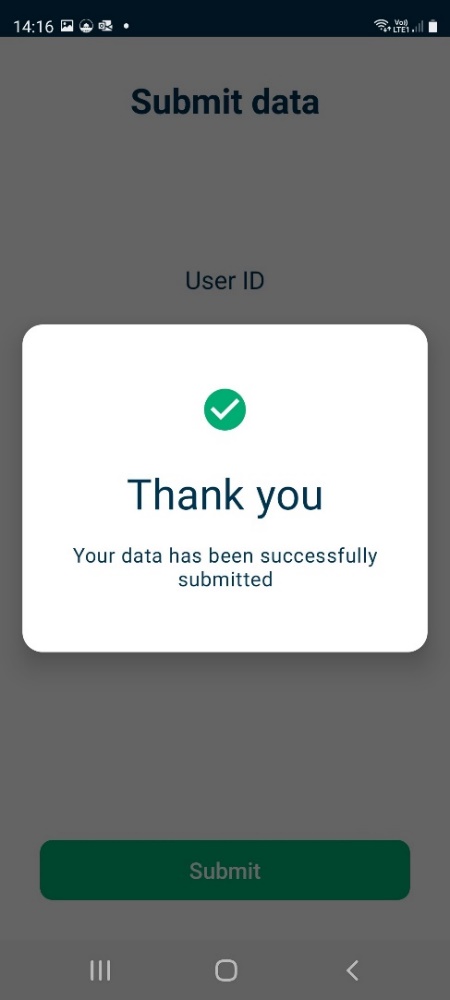 | 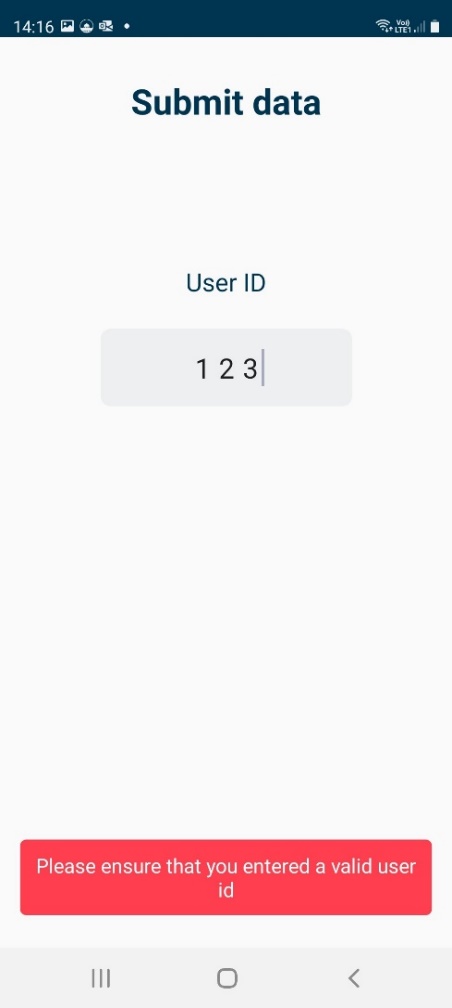 | 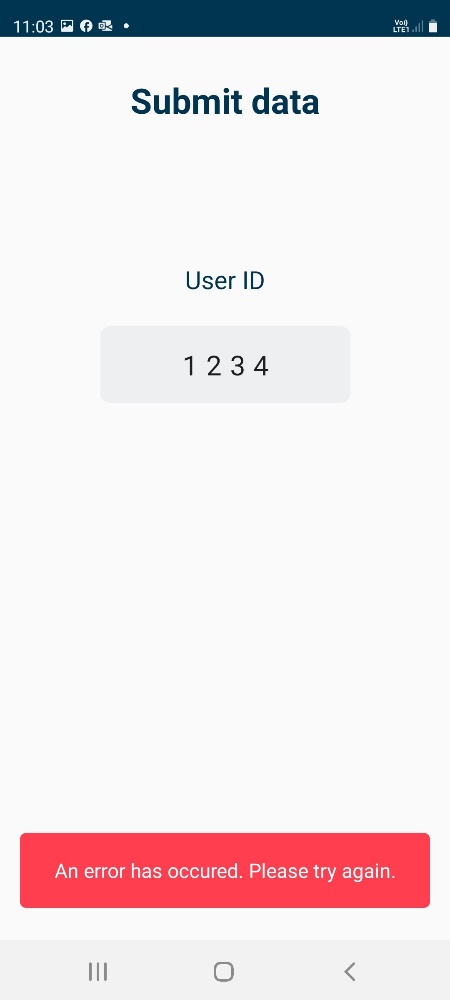 |
| --- | --- | --- |
| C12. Notification for successful report submission | C13. Error notification when you enter invalid user ID | C14. Error notification when you do not have internet connection |

The app does not store any information in your phone, so you will not be able to access your manikin reports once submitted. However, you will receive a report via email of all pain manikin reports at the end of your data collection period of 30 days.


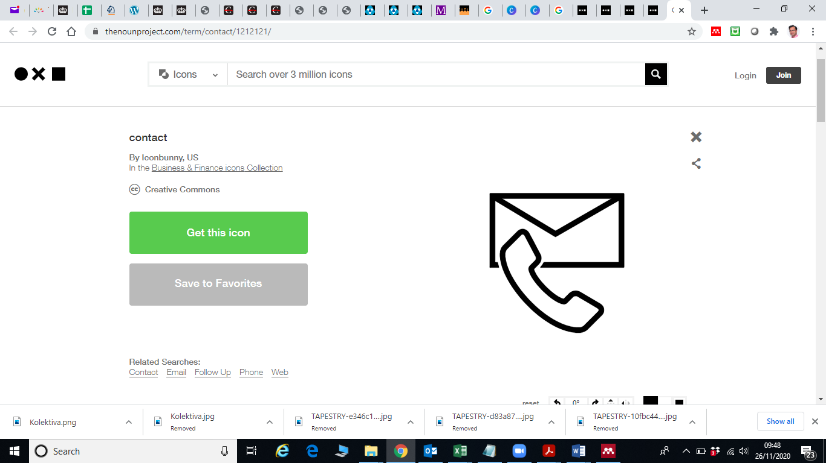


# D. How to get in touch if you need help

**Please contact us if you have**:

- Problems downloading or installing the app on your phone
- Problems connecting your smartphone to the internet
- Problems completing or sending a manikin report
- Forgotten your study ID
- Any questions or concerns about the study in general

**You can contact us**:

1. Any time **via email** by sending us a message **on** [**painmanikin@manchester.ac.uk**](mailto:painmanikin@manchester.ac.uk)

The research team will monitor this email inbox regularly. We will answer your email within two working days.

1. On working days between 8am-6pm **by phone** **on 0745 907 3783**

If your call is not answered, you can leave a message with your contact number and question. A member of research team will get back to you within two working days.

**
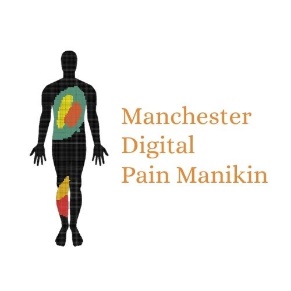
**

We wish you good luck in the study and thank you for your help and patience.


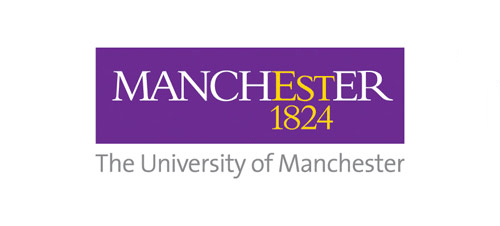

Supplement: sj-docx-2-dhj-10.1177_20552076231194544 - Supplemental material for Feasibility and acceptability to use a smartphone-based manikin for daily longitudinal self-reporting of chronic pain [file sj-docx-2-dhj-10.1177_20552076231194544.docx]
