# Supplementary material for: Feasibility and acceptability to use a smartphone-based manikin for daily longitudinal self-reporting of chronic pain
Source: Digit Health. 2023 Aug 16;9:20552076231194544. doi: 10.1177/20552076231194544 (PMC10434844; doi:10.1177/20552076231194544)
Supplement: sj-docx-3-dhj-10.1177_20552076231194544 - Supplemental material for Feasibility and acceptability to use a smartphone-based manikin for daily longitudinal self-reporting of chronic pain [file sj-docx-3-dhj-10.1177_20552076231194544.docx]

**
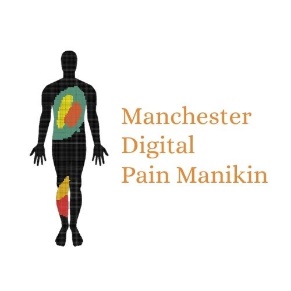
The Manchester Digital Pain Manikin Study**

**Feasibility study**

**End-of-study questionnaire**

**Study ID: _______**

**Instructions for completion:**

Thank you very much for taking out time to complete this survey for taking part in the Manchester Digital Pain Manikin study.

This questionnaire is to ask you 46 questions about the participants’ views on their experience using the MDPM and its acceptability. Completing them will take 15-30 minutes.

You can complete this questionnaire on any digital device, but we advise a tablet, laptop or computer because they have a bigger screen. Please note that if you wish to increase the font size for ease of reading and answering, you can click on the plus sign at the top right hand corner of the questionnaire.

Please feel free to contact the research team at the University of Manchester via email on [painmanikin@manchester.ac.uk](mailto:painmanikin@manchester.ac.uk) if you need assistance. You can also use the contact details on the study flyer.

**End-of-study questionnaire**

| Indicate to what extent you agree with each of the following statements. | | |
| --- | --- | --- |
| 1 | It was easy to complete a pain drawing using the Manchester Digital Pain Manikin app | Strongly disagree  Disagree  Neutral  Agree  Strongly agree |
| 2 | The colours were a good reflection of the different pain intensities | Strongly disagree  Disagree  Neutral  Agree  Strongly agree |
| 3 | Using the Manchester Digital Pain Manikin app was too complicated | Strongly disagree  Disagree  Neutral  Agree  Strongly agree |
| 4 | The Manchester Digital Pain Manikin app included all the important aspects of my pain | Strongly disagree  Disagree  Neutral  Agree  Strongly agree |
| 5 | The pain drawing was a true reflection of my pain | Strongly disagree  Disagree  Neutral  Agree  Strongly agree  Can’t tell |
| 6 | The Manchester Digital Pain Manikin app helped me to report my pain | Strongly disagree  Disagree  Neutral  Agree  Strongly agree  Can’t tell |

**Usability**

| How much do you agree with below statements? Please select one response for each statement. | Strongly disagree | Disagree | Neutral | Agree | Strongly agree |
| --- | --- | --- | --- | --- | --- |
| It was easy to download and install the Manchester Digital Pain Manikin app on my smartphone |  |  |  |  |  |
| Downloading and installing the app does not take long |  |  |  |  |  |
| The user instruction was easy to follow |  |  |  |  |  |
| Understanding how the app works does not take long |  |  |  |  |  |
| It was easy to indicate on the manikin where my pain was |  |  |  |  |  |
| It was easy to select the right pain intensity when drawing my pain on the manikin |  |  |  |  |  |
| Completing one manikin report does not take long |  |  |  |  |  |
| The daily notification helped me to remember to complete a manikin report |  |  |  |  |  |

| How much do you agree with below statements? Please select one response for each statement. | Strongly disagree | Disagree | Neutral | Agree | Strongly agree |
| --- | --- | --- | --- | --- | --- |
| I didn’t mind reporting my pain using the manikin |  |  |  |  |  |
| I found it useful to report my pain using the Manchester Digital Pain Manikin app |  |  |  |  |  |

**Intention to use the Manchester Digital Pain Manikin**

| I would be happy to report my pain using the Manchester Digital Pain Manikin app again for future research studies | Strongly disagree  Disagree  Neutral  Agree  Strongly agree |
| --- | --- |
| For a study that takes longer than one month, how often would you be willing to complete a manikin report? | Multiple times a day  Once a day  Every 2-3 days  Once a week  Less often than once week |
| For how long would you be willing to complete a daily manikin report? | Less than a month  1-3 months  4-6 months  Up to a year  Longer than a year |
| For this study, you received a daily notification to complete your manikin report at [time of notification]. Was this time convenient for you? | Always  Often  Sometimes  Rarely  Never |
| If not, what time would you have preferred (please use a 24-hour format, e.g. 0900, or 1400) | __________________________ |

**Additional features of Manchester Digital Pain Manikin**

| My experience of using the manikin would improve if I could change (tick all that apply): | The gender of the manikin  The skin colour of the manikin  The body shape of the manikin  The language of instructions and questions |
| --- | --- |
| What additional personalisation features you would like to suggest? | _______________________________  _______________________________ |
| Do you have any other suggestions for how we could improve the Manchester Digital Pain Manikin app? | _______________________________  _______________________________ |

**Global Pain Scale**

*Your pain*

**For each question, please indicate your level of pain by selecting a number from 0 to 10**

| My current pain is | No pain 0 1 2 3 4 5 6 7 8 9 10 Extreme pain |
| --- | --- |
| During the past week, the best my pain has been is | No pain 0 1 2 3 4 5 6 7 8 9 10 Extreme pain |
| During the past week, the worst my pain has been is | No pain 0 1 2 3 4 5 6 7 8 9 10 Extreme pain |
| During the past week, my average pain has been | No pain 0 1 2 3 4 5 6 7 8 9 10 Extreme pain |
| During the past 3 months, my average pain has been | No pain 0 1 2 3 4 5 6 7 8 9 10 Extreme pain |

*Your feelings*

**During the past week I have felt:**

| Afraid | Strongly disagree 0 1 2 3 4 5 6 7 8 9 10 Strongly agree |
| --- | --- |
| Depressed | Strongly disagree 0 1 2 3 4 5 6 7 8 9 10 Strongly agree |
| Tired | Strongly disagree 0 1 2 3 4 5 6 7 8 9 10 Strongly agree |
| Anxious | Strongly disagree 0 1 2 3 4 5 6 7 8 9 10 Strongly agree |
| Stressed | Strongly disagree 0 1 2 3 4 5 6 7 8 9 10 Strongly agree |

*Your clinical outcomes*

**During the past week:**

| I had trouble sleeping | Strongly disagree 0 1 2 3 4 5 6 7 8 9 10 Strongly agree |
| --- | --- |
| I had trouble feeling comfortable | Strongly disagree 0 1 2 3 4 5 6 7 8 9 10 Strongly agree |
| I was less independent | Strongly disagree 0 1 2 3 4 5 6 7 8 9 10 Strongly agree |
| I was unable to work (or perform normal tasks) | Strongly disagree 0 1 2 3 4 5 6 7 8 9 10 Strongly agree |
| I needed to take more medication | Strongly disagree 0 1 2 3 4 5 6 7 8 9 10 Strongly agree |

*Your activities*

**During the past week I was NOT able to:**

| Go to the store | Strongly disagree 0 1 2 3 4 5 6 7 8 9 10 Strongly agree |
| --- | --- |
| Do chores in my home | Strongly disagree 0 1 2 3 4 5 6 7 8 9 10 Strongly agree |
| Enjoy my friends and family | Strongly disagree 0 1 2 3 4 5 6 7 8 9 10 Strongly agree |
| Exercise (including walking) | Strongly disagree 0 1 2 3 4 5 6 7 8 9 10 Strongly agree |
| Participate in my favorite hobbies | Strongly disagree 0 1 2 3 4 5 6 7 8 9 10 Strongly agree |

**Global Rating of Change Scale**

Please rate the overall condition of your long-term pain COMPARED TO WHEN YOU FIRST PARTICPATED IN THIS STUDY ONE MONTH AGO (Check only one):

| € A very great deal worse | € About the same | € A very great deal better |
| --- | --- | --- |
| € A great deal worse |  | € A great deal better |
| € Quite a bit worse |  | € Quite a bit better |
| € Moderately worse |  | € Moderately better |
| € Somewhat worse |  | € Somewhat better |
| € A little bit worse |  | € A little bit better |
| € A tiny bit worse |  | € A tiny bit better |

**Reason for non-completion**

| What were reasons for you NOT to complete a manikin report on some days? (tick all that apply) | Not applicable  Forgot  In too much pain  No pain to report  Pain the same as the day before  Technical issues  Other (please specify) |
| --- | --- |
