# Supplementary material for: Feasibility and acceptability to use a smartphone-based manikin for daily longitudinal self-reporting of chronic pain
Source: Digit Health. 2023 Aug 16;9:20552076231194544. doi: 10.1177/20552076231194544 (PMC10434844; doi:10.1177/20552076231194544)
Supplement: sj-docx-4-dhj-10.1177_20552076231194544 - Supplemental material for Feasibility and acceptability to use a smartphone-based manikin for daily longitudinal self-reporting of chronic pain [file sj-docx-4-dhj-10.1177_20552076231194544.docx]

# Supplementary materials

Of the 27 people who consented but did not submit a manikin report, fifteen completed the baseline questionnaire; Table S1 present their characteristics. Of the remaining eight, we had no information on their characteristics available.

Table S1: Characteristics of people who consented to take part and completed a baseline questionnaire, but without submitting a manikin report (n=15)

| **Characteristic** | **Categories** | **Number (percentage)** |
| --- | --- | --- |
| Age | 44 or younger | 3 (20) |
|  | 45 – 64 | 8 (53) |
|  | 65 or older | 4 (27) |
| Gender | Male | 3 (20) |
|  | Female | 12 (80) |
| Ethnicity ^a)^ | White | 11 (73) |
|  | Non-white | 4 (27) |
| Employment status ^b)^ | Employed | 7 (47) |
|  | Not working | 7 (47) |
|  | Other | 1 (6) |
| Index of multiple deprivation decile ^c)^ | 1 to 3 (most deprived) | 7 (46) |
|  | 4 to 6 | 4 (27) |
|  | 7 to 10 (least deprived) | 4 (27) |
|  | Missing | Nil |
| Musculoskeletal condition | Osteoarthritis (OA) | 1 (6) |
|  | Rheumatoid arthritis (RA) | 6 (40) |
|  | Fibromyalgia | 1 (6) |
|  | More than one condition | 4 (28) |
|  | Missing | 3 (20) |
| Pain experience (in years) | Less than a year | 1 (7) |
|  | 1 – 3 years | 6 (40) |
|  | 4 – 10 years | 1 (7) |
|  | More than 10 years | 7 (46) |

a) ‘White’ included all people who identified as being from white ethnic backgrounds, which included mixed/multiple ethnic groups); the remaining ethnicities were recoded as ‘Non-white’

b) ‘Employed’ included employed and self-employed; ‘Not working’ included unemployed, retired, and student. ‘Other’ included, for example, freelancing, and voluntary work

c) The index of multiple deprivation decile is a proxy of socio-economic status (47) derived from postcode, with lower deciles representing more socio-economic deprivation
